# Traits along the leaf economics spectrum are associated with communities of foliar endophytic symbionts

**DOI:** 10.3389/fmicb.2022.927780

**Published:** 2022-07-28

**Authors:** Peter H. Tellez, A. Elizabeth Arnold, Ashton B. Leo, Kaoru Kitajima, Sunshine A. Van Bael

**Affiliations:** ^1^Department of Ecology and Evolutionary Biology, Tulane University, New Orleans, LA, United States; ^2^School of Plant Sciences, University of Arizona, Tucson, AZ, United States; ^3^Department of Ecology and Evolutionary Biology, University of Arizona, Tucson, AZ, United States; ^4^Smithsonian Tropical Research Institute, Panama City, Panama; ^5^Division of Forest and Biomaterial Science, Graduate School of Agriculture, Kyoto University, Kyoto, Japan

**Keywords:** Ascomycota, endophytic fungi, leaf functional traits, symbiosis, tropical forest

## Abstract

Leaf traits of plants worldwide are classified according to the Leaf Economics Spectrum (LES), which links leaf functional traits to evolutionary life history strategies. As a continuum ranging from thicker, tough leaves that are low in nitrogen (N) to thinner, softer, leaves that are high in N, the LES brings together physical, chemical, and ecological traits. Fungal endophytes are common foliar symbionts that occur in healthy, living leaves, especially in tropical forests. Their community composition often differs among co-occurring host species in ways that cannot be explained by environmental conditions or host phylogenetic relationships. Here, we tested the over-arching hypothesis that LES traits act as habitat filters that shape communities of endophytes both in terms of composition, and in terms of selecting for endophytes with particular suites of functional traits. We used culture-based and culture-free surveys to characterize foliar endophytes in mature leaves of 30 phylogenetically diverse plant species with divergent LES traits in lowland Panama, and then measured functional traits of dominant endophyte taxa *in vitro*. Endophytes were less abundant and less diverse in thick, tough, leaves compared to thin, softer, leaves in the same forest, even in closely related plants. Endophyte communities differed according to leaf traits, including leaf punch strength and carbon and nitrogen content. The most common endophyte taxa in leaves at different ends of the LES differ in their cellulase, protease, chitinase, and antipathogen activity. Our results extend the LES framework for the first time to diverse and ecologically important endophytes, opening new hypotheses regarding the degree to which foliar symbionts respond to, and extend, the functional traits of leaves they inhabit.

## Introduction

Plants worldwide host diverse microfungi within healthy leaf tissues. These foliar fungal endophytes, which occur within living leaves without causing disease (Arnold et al., 2000), typically are horizontally transmitted and phylogenetically diverse within individual hosts (Arnold et al., 2009). Some endophytes are latent saprotrophs with limited impact, awaiting leaf senescence (Alvarez-Loayza et al., 2011). Others are latent pathogens, or may be pathogens of co-ocurring plant species (Slippers and Wingfield, 2007). Others still are beneficial, influencing resilience in tropical trees by enhancing defense against pathogens (Arnold et al., 2003; Herre et al., 2007; Mejía et al., 2008), reducing herbivore damage (Van Bael et al., 2009a,b), and altering plant physiology (Westoby and Wright, 2006; Christian et al., 2019).

All tropical trees surveyed thus far host diverse fungal endophytes in their healthy leaves, but predicting their abundance, diversity, composition, and functional roles in natural plant communities can be difficult. In many cases, differences in endophyte communities among co-occurring plants cannot be explained by environmental conditions or the phylogenetic relationships of their hosts (Arnold and Herre, 2003; Arnold and Lutzoni, 2007; Higgins et al., 2011, 2014; Vincent et al., 2016; Oita et al., 2021a,b). This observation suggests that endophytes may be responsive to other factors, such as leaf traits, that may not vary strictly with host plant phylogeny.

Leaf traits of plants worldwide are classified according to the Leaf Economics Spectrum (LES), which links leaf functional traits to evolutionary life history strategies of plants (Wright et al., 2004). On one end of the LES are species that produce long-lived leaves with a high leaf mass per area (LMA, which describes tissue thickness and density), a high investment in mechanical and structural defenses (e.g., leaf toughness and leaf dry-matter content), and low nitrogen (N; Wright et al., 2004; Donovan et al., 2011; Mason and Donovan, 2015; Osnas et al., 2018). On the other end of the LES plant species that produce short-lived leaves with a low LMA, a low investment in mechanical and structural defenses, and high N content (Coley and Barone, 1996; Wright et al., 2004; Donovan et al., 2011; Osnas et al., 2018). Previous studies have linked leaf traits to fungal endophytes in temperate habitats (Saunders et al., 2010; González-Teuber et al., 2020, 2021; Oono et al., 2020), but more research is needed to understand patterns in diverse, tropical forests (e.g., Tellez et al., 2020). In tropical forests, closely related tree species near different ends of the LES often co-occur (e.g., Wright et al., 2004), providing an opportunity to examine the contribution of leaf traits to differences in endophyte assemblages and functional traits at the plant community level.

Here, we test the hypothesis that variation in the abundance, diversity, composition, and functional traits of fungal endophyte communities can be explained by leaf traits as defined by the LES. We examined foliar endophytes in 30 co-occurring species of host plants, drawing from 19 common plant families in a lowland forest in Panama to represent a breadth of traits along the LES (leaf dry matter content, C and N content, LMA, leaf punch strength, and leaf thickness). These included nine species pairs, each comprising closely related species that differ in their LES traits (Table 1; Supplementary Tables S1, S2). This study design allowed simultaneous consideration of leaf functional traits with variables that correlate with endophyte community composition, such as host phylogeny, host identity, and spatial structure (Higgins et al., 2011; Liu et al., 2019). Endophyte communities were assessed with both a culture-based method (all 30 plant species) and a culture-free, high-throughput method (11 species in six families). Finally, strains of the most abundant orders were evaluated for functional traits relevant to their occurrence in leaves, including cellulase, chitinase, protease, and antimicrobial activity. These traits reflect the biotic interactions of horizontally transmitted endophytes with their host plants at the endophytic and saprotrophic phases, and with co-occurring species in the foliar microbiome (see Augspurger and Wilkinson, 2007; dos Reis Almeida et al., 2007; U’Ren and Arnold, 2016).

**Table 1:** Family, species, species code, and number of individuals used in culture-based (*n*_1_) and culture-free (*n*_2_) surveys of fungal endophytes associated with 30 woody plant species in the forest understory of Barro Colorado Island, Panama.

Family	Species	Code	*n*_1_/*n*_2_
Tiliaceae	*Apeiba membranacea*	APEM	3/−
Lauraceae	*Beilschmiedia pendula*	BEIP	3/−
Sapotaceae	*Chrysophyllum argenteum*	CHRA	3/−
Sapotaceae	*Chrysophyllum caimito*	CHRC	1/−
Boraginaceae	Cordia alliodora^*^	CORA	3/2
Boraginaceae	Cordia bicolor^*^	CORB	3/3
Sapindaceae	Cupania rufescens^*^	CUPR	3/−
Sapindaceae	Cupania seemannii^*^	CUPS	3/3
Rubiaceae	*Faramea occidentalis*	FARO	1/−
Clusiaceae	Garcinia intermedia^*^ *^1^*	GARI	3/−
Clusiaceae	Garcinia madruno^*^ *^1^*	GARM	3/−
Lecythidaceae	*Gustavia superba*	GUSS	2/−
Olacaeae	Heisteria acuminata^*^	HEIA	3/3
Olacaeae	Heisteria concinna^*^ *^1^*	HEIC	3/3
Chrysobalanaceae	*Hirtella americana*	HIRA	3/−
Chrysobalanaceae	*Hirtella triandra*	HIRT	2/−
Violaceae	*Hybanthus prunifolius*	HYBP	3/−
Salicaceae	*Laetia thamnia*	LAET	2/−
Piperaceae	Piper cordulatum^*^	PIPC	3/3
Piperaceae	Piper reticulatum^*^	PIPR	3/3
Burseraceae	*Protium panamense*	PROP	2/−
Rubiaceae	Psychotria horizontalis^*^	PSYH	3/2
Rubiaceae	Psychotria limonensis^*^	PSYL	3/−
Fabaceae	*Swartzia simplex* var. continentalis^*^	SWAS	3/3
Fabaceae	*Swartzia simplex* var. grandiflora^*^	SWAC	3/3
Clusiaceae	*Symphonia globulifera*	SYMG	1/−
Combretaceae	*Terminalia amazonia*	TERA	2/−
Malvaceae	*Theobroma cacao*	THEC	2/−
Meliaceae	*Trichilia tuberculata*	TRIT	3/−
Annonaceae	*Xylopia macrantha*	XYLM	2/−

* Denotes tree species used for culture-free surveys.

1 Individuals in these species did not meet the threshold of 4,000 sequences for the culture-free surveys and were excluded from multivariate analyses.

With these data, we tested three main predictions. Because leaf toughness may preclude infection by some endophytes that rely on haustoria or penetration pegs (Huang et al., 2018), and leaf thickness and density could limit intercellular growth (Herre et al., 2007) we predicted that endophyte abundance, diversity, and richness would be lower in thick, tough, and long-lived leaves relative to thin, soft, and shorter-lived leaves. Second, as endophytes are heterotrophs, differences in foliar N or C may support endophytes with distinctive nutritional requirements (U’Ren and Arnold, 2016). We therefore predicted that differences in abundance and composition could be explained by these foliar nutrients. Third, the LES is a defining axis on which life history, ecology, physical structure, and chemistry come together to define leaf traits. We predicted that foliar endophytes would represent an extension of the LES, with the dominant taxa of endophytes differing between leaves at different ends of the LES continuum, and those endophytes in turn differing in traits relevant to leaf function. Together our data show how the LES is associated with foliar symbiont communities and their traits, providing evidence for the underlying mechanisms influencing plant-associated microbial communities that, in turn, shape the structure and function of Earth’s most diverse forests.

## Materials and methods

We completed this study at Barro Colorado Island, Panama (BCI, 9° 9′ N, 79° 51′ W), a former hilltop isolated by the creation of Gatun Lake in 1914 for construction of the Panama Canal (Leigh, 1993). The study area consists of mature forest (> 400 years old) and late secondary forest (90–110 years old; Sagers and Coley, 1995). The region has a mean annual temperature of 27°C and receives an average of 2,600 mm of precipitation annually, primarily in a well-defined wet season that typically begins in May and concludes in December (Leigh et al., 1990).

In June and July of 2015, we collected fresh, mature leaves of 30 phylogenetically diverse woody plant species representing 21 genera and 19 families for culture-based endophyte surveys (Table 1). A subset of 11 species representing six genera was used for culture-free analyses of endophyte communities. We selected co-occurring plant species that represent a range of low to high LMA (Supplementary Table S2; data from S. Joseph Wright, personal communication), are common on BCI, and span a wide phylogenetic breadth of the woody plant diversity at BCI (Kress et al., 2009). Our sampling design included phylogenetic pairing for 18 species, such that in nine cases, two species—one with a lower LMA value and one with a higher LMA value—were selected in the same genus or family.

We collected 15 mature, asymptomatic leaves from each of three individuals per species, for a total of 78 individuals (except when multiple individuals in a given species were not available; Supplementary Table S1). Five leaves per individual were used for measuring leaf structural properties. The remaining 10 leaves per individual were used for measuring leaf mechanical properties, and for culture- and culture-free surveys. We sampled only from individuals with stems >1 cm in diameter at breast height. Focal plants occurred naturally in the forest understory and were sampled opportunistically in mature and secondary forest. Individuals of the same species were collected at localities as distant as possible, ensuring that in general, multiple species were collected in proximity to one another. The average distance between sampled individuals was 0.42 km (range: 0.005–2.10 km) and between conspecifics was 0.97 km (range: 0.41–1.64 km).

Within 24 h of collection, we washed each leaf in running tap water while lightly rubbing off epiphytes and debris. We photographed each leaf next to a ruler for scale prior to functional trait analysis and tissue preparation for surveys of endophytes. We conducted culture-based (isolation of fungi in growth media) and culture-free (Illumina MiSeq) surveys to capture fungi that could be missed by either method (U'Ren et al., 2014); to compare fungal community associations with leaf traits using both survey methods in the event that the communities identified by each approach represented different taxa; and, through culturing, to generate an isolate library for measurements of fungal functional traits, allowing us to test the hypothesis that fungal traits track with the LES.

### Leaf functional trait measurements

We measured structural traits for five leaves per individual following (Pérez-Harguindeguy et al., 2013). For each leaf, we measured fresh weight (FW, g) and dry weight after drying at 70°C for 72 h (DW, g). We calculated leaf dry-matter content (LDMC, g g^−1^) as DW divided by FW. We calculated leaf area (LA, cm^2^) from photographs with Image J (NIH, United States). We calculated LMA (g m^−2^) as LA divided by DW. Dried leaves were analyzed for percent carbon (%C) and nitrogen (%N) at Louisiana State University’s Agricultural Chemistry Laboratory. Associations among leaf traits used for statistical analyses are presented in Supplementary Figure S1.

We measured mechanical traits for 10 leaves per individual following published protocols (Onoda et al., 2008). We used a Mitutoyo 7327 Micrometer Gauge (Mitutoyo, Takatsu-ku, Kawasaki, Japan) to measure leaf thickness (μm) at a single point in the middle of each leaf, taking care to avoid major and secondary veins. To measure leaf punch strength, we used an Imada 2,105 materials testing machine (Imada Inc., Northbrook, IL, United States) to conduct punch-and-die tests with a sharp-edged cylindrical steel punch (2.0 mm diameter) and a steel die with a sharp-edged aperture of small clearance (0.05 mm). This setup allowed us to fracture leaves mainly by shearing, rather than tensile or bending forces (Onoda et al., 2008). Once the punch-and-die test cell was mounted on the testing machine, we set the steel punch so it would pass through the hole of the die without friction. When punching through the leaf, we kept the punch speed constant while the machine simultaneously recorded the maximum load (N) applied. We applied this punch-and-die test to three areas of each leaf: apically, medially, and basally, and averaged measurements for each leaf. Leaf-punch strength (N mm^−1^) was expressed as the maximum load per unit fracture length (i.e., the circumference of the punch). After mechanical trait measurements were taken, the same 10 leaves were used for culture- and culture-free surveys. At this point they were still turgid and fresh.

### Tissue preparation for evaluation of endophyte communities

We removed the tips and margins of each leaf and cut the lamina into 2 mm^2^ segments, which were surface-sterilized by agitating in 95% ethanol (10 s), 10% chlorine bleach [original concentration, 5.25% NaOCl^−^; 2 min], and 70% ethanol (2 min; Higgins et al., 2011). Leaf segments were allowed to surface-dry briefly under sterile conditions prior to further processing for the culture-based and culture-free analyses, below.

### Culture-based survey of endophytes

For isolation of endophytic fungi in culture, 96 surface-sterilized leaf segments per individual tree were placed individually into 1.5 ml microcentrifuge tubes containing 2% malt extract agar (MEA) slants; 0.75 ml MEA/tube and sealed with Parafilm M (Bemis Company Inc., United States). In total, 7,488 tubes were prepared with leaf tissue. We incubated slant tubes at room temperature for 2 months. Over that time, emergent fungal growth was isolated into pure culture on 2% MEA in Petri plates (35 mm diameter). We obtained 2,459 isolates in pure culture. Living vouchers in sterile water were deposited at the Robert L. Gilbertson Mycological Herbarium at the University of Arizona (ARIZ, accession numbers PT0001-PT2459).

We extracted DNA from fresh mycelium (~2 mm^2^) of all isolates with the Extract-N-Amp Plant PCR Kit (Sigma-Aldrich; Sandberg et al., 2014). We used primers ITS1F and LR3 to amplify the nuclear ribosomal internal transcribed spacer region and 5.8S gene (ITSrDNA) and *ca.* 600 bp of the adjacent large ribosomal subunit (LSUrDNA) for a total of *ca.* 1,000 base pairs per sequence (i.e., ITSrDNA-LSUrDNA; Arnold et al., 2007). When amplification failed, we amplified only ITSrDNA with primers ITS1F or ITS5 and ITS4 (U'Ren et al., 2012). PCR products were verified using gel electrophoresis. Positive amplicons were cleaned with ExoSap-IT (Affymetric; Santa Clara, CA, United States) and sequenced bidirectionally with the BigDye Terminator v 3.1 cycle sequencing kit and the original primers on an Applied Biosystems 3730*xl* DNA analyzer (Foster City, CA, United States) at the University of Arizona Genetics Core. We used the ChromaSeq package in Mesquite v. 2.01+ (Maddison and Maddison, 2010) for base-calling and to assemble sequences with *phred* and *phrap* (Ewing and Green, 1998). We used Sequencher v. 5.1 (Gene Codes Corporation, Ann Arbor, MI, United States) to edit contigs manually. Overall, we sequenced ITSrDNA or ITSrDNA-LSUrDNA successfully for 1,870 isolates (76% of isolates).

We used the Tree-Based Alignment Selector (T-BAS) toolkit to determine phylogenetic placement of isolates within the Pezizomycotina (Ascomycota), to visualize relationships and metadata in a phylogenetic context (Miller et al., 2015; Carbone et al., 2017), and to designate operational taxonomic units (OTUs) on the basis of sequence similarity (95, 97, and 99% sequence similarity). Groups based on 95% sequence similarity approximate species boundaries in focal taxa of tropical endophytes and were used to define OTUs for statistical analyses (U'Ren et al., 2009; Oita et al., 2021a,b). Results for analyses with 97 and 99% similarity groups were comparable (data not shown) but had more singleton OTUs.

We estimated endophyte abundance (i.e., isolation frequency) as the proportion of 96 tissue segments that yielded an endophyte in culture (Sandberg et al., 2014). We calculated endophyte diversity as Fisher’s alpha (hereafter, diversity), which is robust to variation in sample size (Fisher et al., 1943). We used Kruskal–Wallis tests to examine differences in endophyte abundance and diversity among host species and genera. A total of 23 host individuals were included in measures of abundance at the community level but excluded from diversity assessments and multivariate analyses because of low isolation frequency. We excluded one host individual as an outlier, due to endophyte diversity value >2 SDs from the mean. From subsequent analyses, we excluded three *Hirtella americana* individuals for which we could not collect leaf thickness data due to an abundance of leaf trichomes that prevented accurate measures. Ultimately, our culture-based data set for statistical analyses included 51 host individuals from 24 plant species, representing 18 genera and 19 families.

### Culture-free survey of endophytes

For culture-free analyses we focused on congeneric or confamilial species pairs, with each pair consisting of one tree species with low LMA and one species with a high LMA (Supplementary Table S2). Tissue processing and DNA extraction followed (U'Ren et al., 2014). Briefly, 96 tissue segments processed per above were placed into sterile CTAB (1 M Tris–HCl pH 8, 5 M NaCl, 0.5 M EDTA, and 20 g CTAB) and stored in sterile 1.5 ml microcentrifuge tubes for up to 2 months at −80°C prior to processing. Four tubes were prepared per individual (each containing 24 tissue segments and 1 ml of CTAB). To prepare for DNA extraction, we decanted the CTAB from each tube, added 0.5 g of sterile beads (5/23″ stainless steel, OPS Diagnostics, Lebanon, NJ, United States), immersed each tube in liquid nitrogen for 4 min, and then used a Next Advance Bullet Blender Storm bead beater to homogenize leaf tissue to a fine powder. We used the MoBio PowerPlant Pro DNA extraction kit (Molecular BioProducts Inc., San Diego, CA, United States) to extract total genomic DNA, which we quantified by Qubit Fluorometric Quantification 2.0 (Thermo Scientific, Wilmington, DE, United States). Extraction products for each host individual (i.e., four tubes) were combined prior to PCR (U'Ren et al., 2019).

Methods for PCR and sequencing followed the two-step amplification process described by Sarmiento et al. (2017); Daru et al. (2019), and U’Ren et al. (2019). Briefly, in an initial PCR we amplified ITSrDNA with primers that contained a universal 22 basepair (bp) consensus sequence tag (CS1 forward, CS2 reverse), 0–5 bp for phase-shifting, a 2 bp linker, and primers (ITS1F and ITS4). We ran the initial PCR in triplicate with 15 μl reactions: 0.5 μl DNA template, 7.5 μl 1X Phusion Flash High-Fidelity PCR Master Mix (ThermoFisher Scientific, Austin, TX, United States), 0.2 μl of 50 μM forward and reverse primers, 1 mg/ml of molecular grade bovine serum albumin (BSA; New England Biolabs, Ipswich, MA, United States), and 6.0 μl of molecular grade water. We used sterile, molecular grade water rather than DNA template for PCR negative controls, and included extraction blanks for each extraction kit. Cycling parameters, electrophoresis, and quantification followed Sarmiento et al. (2017) (see also U’Ren and Arnold, 2017). We used 1 μl of PCR product from samples and negative controls as template for a second PCR set, pooling three initial PCR products for each sample and then diluting 5 μl of the pooled amplicons with molecular grade water to a final concentration of 1:15 (Sarmiento et al., 2017; U’Ren and Arnold, 2017). Each PCR reaction (20 μl) contained 1X Phusion Flash High-Fidelity PCR Master Mix, 0.075 μM barcoded primers (forward and reverse pooled at a concentration of 2 μM), and 0.24 mg/ml of BSA. Cycling parameters, electrophoresis, and quantification followed Sarmiento et al. (2017) and U’Ren and Arnold (2017).

We normalized amplicons to 1 ng/μl and pooled 2 μl of each for sequencing. To purify amplicons we used Agencourt AMPure XP beads (Beckman Coulter, Inc., Brea, CA, United States) at a ratio of 1:1, following the manufacturer’s instructions. Products were evaluated with a BioAnalyzer 2100 (Agilent Technologies, Santa Clara, CA, United States) prior to sequencing.

We did not detect any contamination visually or by fluorometric analysis, but for robust controls we combined 5 μl of each PCR negative and extraction blank, processed them as above, and sequenced them with our samples. We followed protocols of the IBEST Genomics Core at the University of Idaho for paired-end sequencing on the Illumina MiSeq platform with Reagent Kit v3 (2 × 300 bp).

All molecular work for the culture-free survey was done with sterile, aerosol-resistant pipette tips with filters. We used separate reagents, pipettes, tips, and consumables for pre- and post-PCR setup. We prepared PCR mixes in a sterile laminar flow hood that was never exposed to amplified DNA. We decontaminated all surfaces and tools with DNA Away (Molecular BioProducts Inc., San Diego, CA, United States) and treated all surfaces with UV light for 30 min prior to each use (Sarmiento et al., 2017; U’Ren and Arnold, 2017). A second sterile hood was used for pooling and dilutions of the initial PCR products, preparation of the second PCR set, and amplicon pooling (Sarmiento et al., 2017). We used a phylogenetically diverse mock community to ensure that all major lineages of fungi and of the Pezizomycotina could be observed if present (for details see Daru et al., 2019).

Post-sequencing processing (demultiplexing, quality assessment, selection of the appropriate length cutoff, removal of singletons, and designation of OTU) followed Sarmiento et al. (2017). OTUs from negative controls were removed from the data set prior to analysis in order to provide the most stringent quality control for the data set. From the 11 plant species chosen for culture-free surveys, the final data set included 639,852 high-quality fungal sequences with 17,773 ± 17,128 (mean ± SD) sequences per individual plant. Rarefaction curves demonstrated that sequencing depth was sufficient at 4,000 sequences to capture species richness (Supplementary Figure S2), consistent with previous studies (e.g., rarefaction ranging from 950 to 4,000 sequences: Kembel and Mueller, 2014; Laforest-Lapointe et al., 2016). Seven trees in three species did not meet that threshold and were excluded from statistical analyses. After rarefaction, 556,365 sequences remained in our culture-free survey, representing 29 individuals in 11 plant species and six families.

### Functional trait characterization for endophytes

We evaluated functional traits of representative isolates to link characteristics of endophytes with those of their hosts along the LES. The most abundant orders obtained in culture differed in their affiliations with leaves along the LES: Xylariales were isolated most frequently from leaves that were low in %C and high in %N (see Results), whereas Botryosphaeriales were isolated most frequently from leaves that were high in %C and low in %N (see Results). We selected 106 total strains (77 strains of Xylariales, 29 strains of Botryosphaeriales; Supplementary Table S3) for measurements of four traits relevant to leaf characteristics: cellulase activity, chitinase activity, protease activity, and antipathogen activity. *In vitro* degradation of cellulose, chitin, and proteins is strongly associated with antimicrobial defense by plant-associated fungi (dos Reis Almeida et al., 2007; Orlandelli et al., 2015; Halo et al., 2019). Moreover, cellulose is the major component of plant cell walls and is degraded by endophytes during the infection process and/or during the portions of enodphyte life cycles that occur in leaf litter (U’Ren and Arnold, 2016). Cellulose also is an important cell wall component of Oomycete pathogens that are common pathogens of tropical forest trees (Augspurger and Wilkinson, 2007). Chitin occurs in the cell walls of fungi and diverse proteins are often expressed during the infection cycle by pathogens. Methods for cellulase, chitinase, and protease assays followed Gazis et al. (2012) and Bascom-Slack et al. (2012). Cultures were initiated on assay plates and observed at room temperature and natural light–dark cycles for up to 4 weeks. Plates were scored for presence of activity (clearing, cellulase, and protease assays) or color changes (chitinases) per each protocol (Supplementary Table S3). Each assay included positive and negative controls to validate observations. We used logistic regression to evaluate the probability of activity each assay.

These indirect measures of antipathogen activity were complemented by direct dual-culture antagonism assays of focal endophytes with a pathogenic strain of *Fusarium* sp. (AEA-1), which causes disease in seeds and leaves of diverse tropical trees at BCI (Shaffer et al., 2016; Zalamea et al., 2018). We selected 17 representative strains that displayed both chitinase and protease activity (11 strains) or neither chitinase nor protease activity (six strains) for dual-culture antagonism assays (Supplementary Table S4). The endophytes included nine strains of Xylariales and seven strains of Botryosphaeriales. Assays followed Del Olmo-Ruiz (2012), except that plates were scored after 5 days and percent inhibition was calculated for both the pathogen and endophyte in each pairing. Percent inhibition values were evaluated by *t*-tests. Tests of self-inhibition were included to differentiate antipathogen activity from nutrient competition (no self-inhibition was detected).

### Statistical analyses

We used partial least squares regression (PLSR) to evaluate the importance of leaf functional traits on measures of endophyte abundance, diversity, and richness. PLSR is increasingly used in microbial ecology (Stepanauskas et al., 2003; Allen et al., 2005; Bokulich et al., 2014; Peralta et al., 2016; Attermeyer et al., 2017) to assess variation in microbial communities as a function of multiple predictor variables that are often non-independent and intercorrelated (Wold et al., 2001; Carrascal et al., 2009; Attermeyer et al., 2017). PLSR reduces a set of intercorrelated explanatory variables into uncorrelated latent variables (LV) that have maximum covariance with the response variable. We determined the number of LV to retain in the PLSR model based on the leave-one-out (LOO) cross validation method, retaining the two LV with the lowest root mean squared error of prediction (RMSEP) value generated from six components (Peralta et al., 2016). We used three separate PLSR analyses to determine the association of endophyte abundance, diversity, and richness (response variables) to leaf functional traits (predictor variables).

For PLSR model interpretation, we present loading plots that illustrate the correlation structure of the predictor variables in explaining variation in response variables. Associations among variables can be extracted from the spatial distribution of the loading plot: predictor variables that are projected negatively along the *x*-axis are negatively associated with variability in the response variables, and projected positively along the *x*-axis when positively associated with that variability (Allen et al., 2005; Attermeyer et al., 2017). We also present variable importance on projections (VIP), which summarizes the importance of the predictor variables: variables with values >1 are highly influential in the model, 1.0–0.7 are moderately influential, and <0.7 are less influential (Attermeyer et al., 2017). Before analyses, predictor and response variables were mean-centered and scaled to unit variance (Attermeyer et al., 2017).

For the culture-based and culture-free surveys, we used distance-based redundancy analyses (dbRDA) to visualize associations between leaf functional traits and endophyte community composition. We excluded OTUs represented by a single sequence in the culture-based data set (singletons) and calculated a Bray–Curtis dissimilarity matrix based on endophyte relative abundance data. We used LMA, LDMC, leaf punch strength, leaf thickness, %C, and %N as the constraining variables. Next, we used permutational multivariate ANOVA (PERMANOVA) marginal tests to assess the amount of variation in endophyte community composition explained by each leaf trait. We used Nonmetric Multidimensional Scaling (NMDS) to visualize patterns in endophyte communities among host taxa. We tested for differences in endophyte community composition as a function of host species and genus by using PERMANOVA with 999 permutations (Anderson and Walsh, 2013). We used a permutational analysis of multivariate dispersion (PERMDISP; Anderson and Walsh, 2013) to test for homogeneity of variance in endophyte communities. We used a PERMDISP as a companion to PERMANOVA to exclude the possibility that any significant differences were caused by differences in the variance of endophyte communities. The relative explanatory power of host species and leaf functional traits on endophyte community composition was compared using a partial-dbRDA.

We used Mantel tests to test for associations between endophyte community composition and host spatial structure and host phylogenetic relatedness. We also used a Mantel test to examine the association between host phylogenetic relatedness and leaf functional traits, though we found no correlation (*p* = 0.87, *r* = 0.01). Host phylogenetic relationships were based on estimates of branch length among host species based on a previously published maximum likelihood phylogeny for woody plant species from BCI (Kress et al., 2009).

We used PLSR to model associations among leaf functional traits and fungal taxonomic orders. The resulting PLSR projection detected associations among fungal orders Xylariales and Botryosphariales and leaf functional traits (Supplementary Figure S3). We used these fungal orders for further examination using Pearson correlation coefficient and scatterplots to test and visualize relationships between specific fungal orders and leaf functional traits. We performed all multivariate analyses using the *pls* (Wehrens and Mevik, 2007), *ape* (Paradis et al., 2004), *picante* (Kembel et al., 2010), and *vegan* (Oksanen et al., 2007) packages in R 3.1.1 (R Team, 2000). Analyses for functional traits of fungi are described above, with analyses for these assays conducted in JMP v14 (SAS Institute, Cary, NC, United States).

## Results

**Figure 1:**
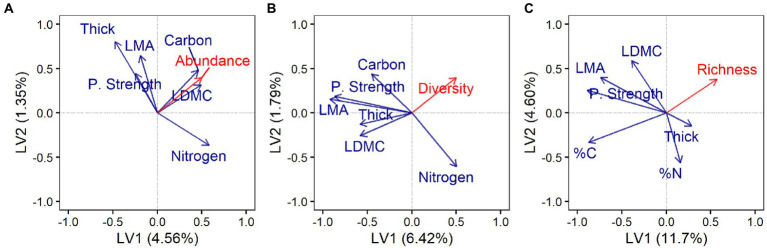
Endophyte abundance, diversity, and richness varied with leaf traits along the leaf economics spectrum. First two latent variables (LV) from partial least squares regression (PLSR) showing correlations between leaf functional traits (blue) and **(A)** endophyte abundance (culture-based survey; 24 host species), **(B)** endophyte diversity (culture-based survey; 24 host species), and **(C)** endophyte richness (culture-free survey; 11 host species). Axis labels indicate the variance in endophyte response explained by each LV. Model description and output can be found in the section “Materials and Methods,” Supplementary Tables S5, S6. Labels: Leaf mass per area (LMA); leaf dry-matter content (LDMC); leaf punch strength (P. Strength); leaf thickness (Thick); percent leaf carbon content (%C); and percent leaf nitrogen content (%N).

Endophytes were isolated on average from 44% ± 22 (mean ± SD) of 96 leaf segments per each individual host. Culture-based endophyte abundance did not differ significantly among tree species or genera when traits along the LES were not considered explicitly (Supplementary Table S5). Instead, as predicted, endophyte abundance was associated negatively with LMA, leaf punch strength, and leaf thickness (Figure 1A; but only leaf punch strength was highly influential in the model; Supplementary Table S6), and positively with leaf dry-matter content (LDMC), %C, and %N (Figure 1A; all highly influential; Supplementary Table S6).

From each host individual, we observed an average of 14.5 ± 7.28 (mean ± SD) species of endophytes in the culture-based survey (Supplementary Table S1). In the culture-based survey, the majority of endophytes for which sequence data were obtained were Ascomycota (1,856 of 1,870 strains), comprising 286 OTUs. A total of 136 OTUs were represented by only one isolate (i.e., were singletons; 47.6%). Cultures represented Sordariomycetes (70.3%), Dothideomycetes (18.1%), Eurotiomycetes (9.1%), and other classes (2.5%), with Xylariales (Sordariomycetes) and Botryosphaeriales (Dothideomycetes) isolated most frequently.

From the culture-free survey, we observed an average of 111.7 ± 82.4 (mean ± SD) species of endophytes per host individual (Supplementary Table S1). We detected 2,172 fungal OTUs (defined by 95% sequence similarity) in the leaves of 11 plant species. After rarefaction, we obtained 1,701 OTUs from 29 host individuals of 11 plant species representing six families. Singletons comprised 384 (22.57%) of the total dataset after rarefaction. Observed OTUs (Richness) ranged from 28 to 373 (111.76 ± 82.40 OTUs per species; mean ± SD) and did not differ significantly as a factor of host species or genus. Phylogenetic placement indicated that the dominant endophyte classes were Sordariomycetes (41.4%) and Dothideomycetes (35.2%), followed by Euromycetes (21.7%), and other lineages (Saccharomycetes, Lichinomycetes; 1.6%).

Diversity and richness did not differ significantly among host species or genera when traits along the LES were not considered explicitly (Supplementary Table S5). Instead, diversity of endophytes isolated in culture was associated negatively with LMA, LDMC, leaf punch strength, leaf thickness, and %C (Figure 1B; LMA, LDMC, and leaf punch strength were highly influential; Supplementary Table S6), and positively with %N (moderately influential; Figure 1B; Supplementary Table S6). Richness of endophytes observed *via* the culture-free approach was associated negatively with LMA, leaf punch strength, and %C (Figure 1C; all highly influential in the model; Supplementary Table S6).

**Figure 2:**
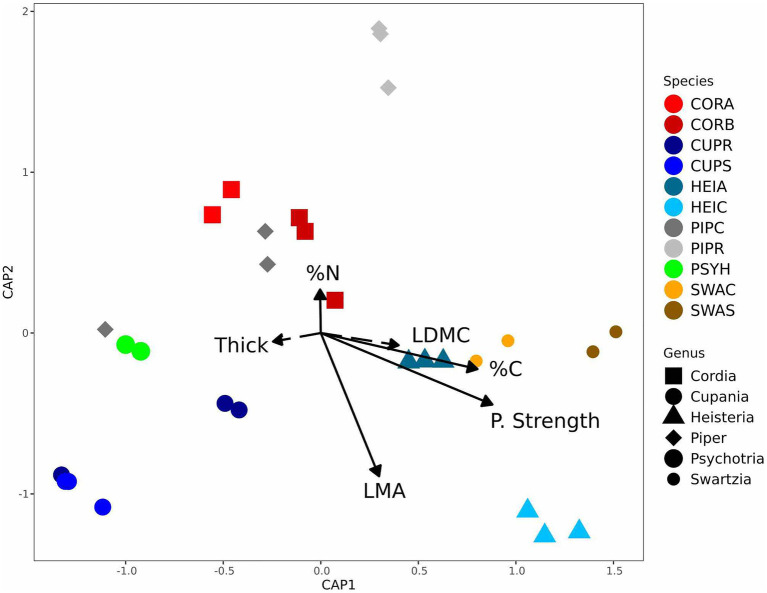
Endophyte community composition was associated with leaf traits from the leaf economics spectrum (culture-free data set). Endophyte community variation within and between 11 host species in six genera (*n* = 29), from distance-based redundancy analyses (dbRDA) models constrained by leaf traits. Solid arrows represent significant associations (*p* < 0.05). Each point represents an endophyte community sampled from one host tree; colors represent host species; and symbols represent host genus (for a list of host species abbreviations, see Table 1).

Endophyte community composition differed significantly among host species and genera in both the culture-based and culture-free surveys (Supplementary Figures S4, S5; Table 2); and endophyte community heterogeneity differed significantly for host species in the culture-based surveys as well. Moreover, variation in endophyte community composition was not associated with phylogenetic relatedness or spatial proximity of hosts (Supplementary Table S7). Instead, differences in endophyte community composition were associated significantly with LMA, leaf punch strength, %C, and %N (Figure 2; Supplementary Figure S5) in both the culture-free and culture-based surveys (Supplementary Table S8). LMA and leaf punch strength explained the most variation followed by %C and %N (Supplementary Table S8). Variance in endophyte community composition explained by leaf traits in the culture-based and culture-free survey was 10 and 8.7%, respectively, when controlling for host species (Supplementary Table S9); when controlling for leaf traits, host species explained 48 and 42% of variance in endophyte community composition in the culture-based and culture-free survey, respectively (Supplementary Table S9).

**Table 2:** Endophyte community composition differed among host taxa.

Factors	PERMANOVA			PERMDISP	
	*F*	*p*	*R^2^*	*F*	*p*
*Culture-based*					
Species	1.36	**0.002**	0.55	2.16	**0.032**
Genus	1.38	**0.003**	0.41	2.01	0.061
*Culture-free*					
Species	4.68	**<0.001**	0.72	1.82	0.108
Genus	3.30	**<0.001**	0.41	1.40	0.250

Variance in endophyte community composition (PERMANOVA) and endophyte community heterogeneity (PERMDISP) explained by host taxonomy at the level of species and genus. PERMDISP is a multivariate test for equality of variance. Results are for the culture-based and culture-free surveys. Bold numbers show *p*-values < 0.05.

**Figure 3:**
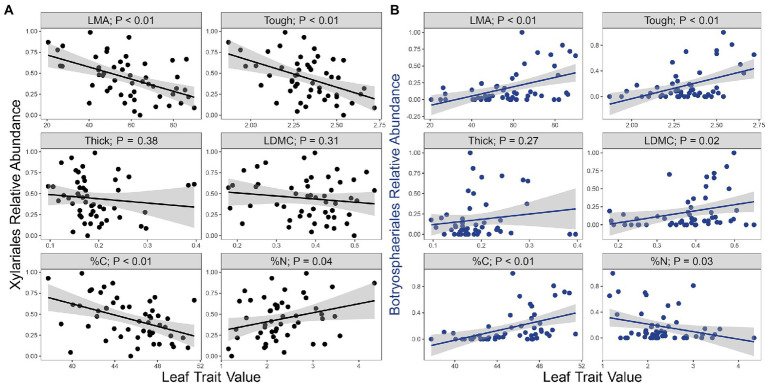
The two most common fungal orders showed contrasting relationships with leaf functional traits. Associations between the relative abundance of fungal orders **(A)** Xylariales (Sordariomycetes) and **(B)** Botryosphaeriales (Dothideomycetes) to leaf mass per area (LMA, g m^−2^), and leaf punch strength (“Tough,” log N mm^−1^), leaf thickness (Thick, log μm), leaf dry matter content (LDMC, g g^−1^), leaf carbon (%C, log %), and leaf nitrogen (%N, log %). Grey bands represent the SEs of the regression lines. Analyses are from the culture-based survey (*n* = 51).

Such community variation as a function of leaf traits reflects in part the abundance of the two most commonly encountered orders in the culture-based survey, Xylariales and Botryosphaeriales (Supplementary Figure S6). Endophytes representing Xylariales were associated negatively with %C, LMA, LDMC, and leaf punch strength, but positively with %N (Figure 3A; Supplementary Table S10). The relative abundance of Botryosphaeriales followed opposite patterns (Figure 3B; Supplementary Table S10). As such, the relative abundance of Xylariales was negatively associated with the relative abundance of Botryosphaeriales (Supplementary Figure S7). We observed no consistent evidence of mutual antagonism between these fungi in dual culture assays (Arnold, unpublished), suggesting that their distributions reflect leaf traits rather than direct fungal interactions.

Strains of Xylariales were significantly more likely to degrade cellulose, chitin, and protein than strains of Botryosphaeriales (Fisher’s exact test in each case, *p* = 0.0012, *p* = 0.0102, and *p* < 0.001, respectively; Supplementary Table S3). In dual culture assays with a representative pathogen, Xylariales were significantly more inhibitory toward the pathogen (*t* = 2.41, *df* = 15, *p* = 0.0295), and tended to be less inhibited by the pathogen relative to Botryosphaeriales (*t* = −1.60, *df* = 15, *p* = 0.0652; Supplementary Table S4). When these assays were linked, endophytes with both chitinase and protease activity inhibited the pathogen significantly more than endophytes with neither activity (*t* = −2.07, *df* = 15, *p* = 0.0280) and were less inhibited by the pathogen in turn (*t* = 1.91, *df* = 15, *p* = 0.0377, Supplementary Table S4). Thus the most common orders, which affiliated at distinctive relative abundances in leaves with different traits along the LES, exhibit robust functional differences themselves.

## Discussion

The Leaf Economics Spectrum (LES) showcases the linkages among life history traits as they manifest in leaves worldwide. Here, we examined how the LES extends to the highly diverse foliar symbionts—endophytes—that occur in leaves of all plants studied thus far. In the context of tropical forests, where plant- and endophyte diversity typically reach their peaks, we tested the hypothesis that leaf functional traits as defined by the LES can explain patterns of fungal endophyte abundance, diversity, community composition, and traits. Our culture- and culture-free surveys of phylogenetically diverse tree species in lowland Panama revealed strong associations between leaf traits and endophyte assemblages. Our assays show that the most common endophytes that affiliate with leaves at different ends of the LES differ in their own traits in a predictable manner. Our results extend the LES framework to diverse and ecologically important foliar fungal symbionts and highlight that endophytes may not only respond to, but potentially extend, the functional traits of leaves they inhabit (e.g., in terms of antipathogen defense). Here, we examine three key results and their implications.

### Perspectives on mechanical and structural elements of leaves

We predicted that endophyte abundance, diversity, and richness would be lower in thick, tough, and long-lived leaves relative to thin, soft, and shorter-lived leaves. We found negative associations of endophyte abundance, diversity, and richness with leaf punch strength (toughness) and LMA (density; Figure 1) suggest that the mechanical and structural properties of leaves modulate the form and function of foliar symbioses. In plant-herbivore associations, structural leaf features (e.g., specific leaf weight, lamina, and cuticle thickness) often correlate negatively with densities of herbivorous insects (Peeters, 2002), presumably due to physical feeding limitations imposed by these leaf traits. Similarly, leaf punch strength is a mechanical trait that is a key defense against certain insect herbivores (Coley, 1988; Nichols-Orians and Schultz, 1989; Kursar and Coley, 2003) and is linked to lower diversity in herbivore communities (Peeters, 2002).

The negative association we see between such leaf traits and endophytes may follow similar patterns, albeit due to different mechanisms. For instance, it has been hypothesized that increases in the structural and mechanical properties of leaves of slow-growing plant species may impede or limit endophyte entry and hyphal extension in leaves (Sanati Nezhad and Geitmann, 2013), with fungal entry being easier in thinner, less dense leaves (Arnold and Herre, 2003). Additionally, our results indicated that endophyte abundance, diversity, and richness had a small positive correlation to %N in leaves. This suggests that fast-growing plant species might produce N-based nutrients (e.g., amino acids) or other N-based resources that support higher densities of fungi, much like greater nutrition in leaves is expected to support greater abundances of insect herbivores (Cornelissen and Stiling, 2006; Poorter and Bongers, 2006). Taken together, our results suggest that structural, mechanical, and chemical properties of leaves influence the type and number of fungal taxa able to colonize a leaf’s interior.

### Perspectives on variation in endophyte community composition across leaves on the LES

We predicted that differences in endophyte community composition could be explained by traits such as LMA, C, and N. LMA, leaf punch strength, and %C and %N content were small but significant contributors to variation in endophyte community composition (Figure 2). These leaf traits are indicators of plant function and life history strategy (Wright et al., 2004, 2010). LMA reflects tradeoffs in allocating resources for either metabolic mass (i.e., mesophyll cytoplasm) vs. cell wall (Agrawal and Fishbein, 2006; Osnas et al., 2018). Leaf mechanical strength and relative abundance of cell wall fiber covary with LMA and often mirror investments in leaf defense and longevity (Kitajima et al., 2013, 2016). Tougher leaves with high LMA and cell wall fiber contents (in particular, cellulose, which has tensile strength higher than steel on a mass basis) may create significant impediment to extension of fungal hyphae. Another dense tissue, i.e., epidermal cuticle, increases its relative contribution to LMA in shade leaves, and thick cuticles may resist punching by hyphal penetration pegs. A previous study in temperate forests found that variation in cell wall polysaccharides correlated with unique fungal endophyte communities (González-Teuber et al., 2020). Collectively, these cell-wall associated structures may act as environmental filters to assemble endophyte communities.

As anticipated, endophyte community composition did differ as a function of %C and %N in leaves, suggesting differences in nutrient use among the major groups of endophytes that occur in leaves at different points along the LES. We found associations between %N and endophyte communities, indicating that important N-based physio-chemical components (e.g., alkaloids or nutrients) in leaves may have some effects on endophyte communities. This is and may interact with physiological components found within cells. In another recent study, Christian et al. (2020) showed correlations between host plant secondary chemistry and fungal endophyte communities in the alkaloid-rich genus, *Psycotria*. In their study of temperate trees, however, González-Teuber et al. (2020) found that fungal endophyte communities in temperate trees were associated with C-based compounds, such as anthocyanins, flavinoids, and terpenoids.

Together with previous studies, our findings point to leaf structural and chemical components as significant contributors shaping endophyte communities in tropical forests (Christian et al., 2020; González-Teuber et al., 2020), extending work on foliar endophytes of 11 species in Papua New Guinea (Wold et al., 2001; Vincent et al., 2016) and complementing the work of Kembel and Mueller (2014) who linked leaf-surface fungal communities in 51 tree species in Panama to LMA and N content (but not C content). This study builds upon these previous efforts by quantifying leaf traits from which endophytes were obtained, and using a combination of culture-based and culture-free surveys to capture the full diversity of endophytes. Our study adds to the growing body of literature indicating functional traits of tropical plants act as a filter to shape communities of highly diverse foliar symbionts that, in turn, have strong effects on plant physiology, productivity, and demography (Arnold et al., 2003).

### Perspectives on traits of endophytes

We found support for the prediction that dominant fungal taxa, Xylariales and Botryosphaeriales, would differ between leaf traits at different ends of the LES continuum (Figure 3), and in turn these taxa would differ in traits relevant to leaf function. The robust bioactivity of endophytic Xylariales, which included cellulase, chitinase, protease, and antipathogen activity, is consistent with a potentially defensive role in the softer, lower-C leaves with limited structural protection in which they most frequently occur. Recent work has highlighted the remarkable chemical diversity of Xylariales such that the traits observed in this study may be relevant beyond the specific strains studied here (Franco et al., 2022). Notably, some strains of Botryosphaeriales also displayed bioactivity, but in general these traits were less common, consistent with their occurrence in leaves with robust structural defenses. That the relative abundance of Xylariales and Botryosphaeriales were negatively associated also raises the question of the importance of direct or indirect interactions among endophytes, especially the roles fungi may play in impeding leaf colonization of other fungal taxa. This is a topic for further research.

Our study is novel in showing that variation in endophyte traits themselves, and their interactions with host plants, one another, and other co-occurring organisms, may underlie the variance in endophyte abundance, diversity, community composition, and traits that remains to be explained even when the LES is taken into account. We found that host identity had a strong effect on endophyte community composition (Table 1), consistent with previous research in the tropics, e.g., ferns of Costa Rica (Del Olmo-Ruiz and Arnold, 2014); tree species of Papua New Guinea (Vincent et al., 2016); tropical trees in Panama (Arnold and Lutzoni, 2007); endemic plant species of Peru (Unterseher et al., 2013); and seeds of lowland tropical trees (Sarmiento et al., 2017). We suggest that other foliar traits related to host identity (e.g., phenology, secondary metabolites, and physiology) may be important in explaining the variance in endophyte communities, either directly (by selecting for individual endophyte taxa) or indirectly (by providing a context for selection of cohorts of associated endophyte taxa that coexist successfully within the same leaf tissues, with compatible sets of functional traits).

## Conclusion

By identifying key traits of the Leaf Economics Spectrum associated with foliar fungal communities, we begin to uncover the mechanisms affecting endophyte community assembly. Previous studies have focused primarily on dispersal limitation and abiotic habitat filters (Saunders et al., 2010), but the topic of leaf traits as host-imposed habitat filters that may act directly on fungi, and shape fungal-fungal competition to act indirectly, has remained relatively underexplored. Our study highlights the need for a trait-based approach to bridge this knowledge gap. The microbiota associated with plants and animals are linked to host health, performance, survival, and evolution (Rosenberg and Zilber-Rosenberg, 2016; Lutzoni et al., 2018). Foliar mycobiomes are likely to act as an extended plant phenotype that protects photosynthetic tissues against abiotic stressors and natural enemies (Ganley et al., 2004; Mejía et al., 2008, 2014; Van Bael et al., 2009a). Considered together, our conclusions suggest that the interplay between plant functional traits and host identity play an important role in shaping the distribution of microbial endophyte communities in tropical forests, with implications that scale up to the shaping the function and dynamics of Earth’s richest forest ecosystems.

## Data availability statement

The datasets presented in this study can be found in online repositories. The names of the repository/repositories and accession number(s) can be found at: https://www.ncbi.nlm.nih.gov/, SAMN14605627-SAMN14605636 and ON153 232–ON155426.

## Author contributions

SV, AEA, and KK designed research. PT, AL, AEA, and SV performed research. PT, SV, and AEA analyzed data and wrote the paper with input from all authors. All authors contributed to the article and approved the submitted version.

## Funding

This research was funded by NSF DEB-1556583 to SV and NSF DEB-1556287 to AEA, and by the School of Plant Sciences and the College of Agriculture and Life Sciences at The University of Arizona (AEA).

## Conflict of interest

The authors declare that the research was conducted in the absence of any commercial or financial relationships that could be construed as a potential conflict of interest.

## Publisher’s note

All claims expressed in this article are solely those of the authors and do not necessarily represent those of their affiliated organizations, or those of the publisher, the editors and the reviewers. Any product that may be evaluated in this article, or claim that may be made by its manufacturer, is not guaranteed or endorsed by the publisher.

## Supplementary material

The Supplementary materials for this article can be found online at: https://www.frontiersin.org/articles/10.3389/fmicb.2022.927780/full#supplementary-material
