# Supplementary material for: Traits along the leaf economics spectrum are associated with communities of foliar endophytic symbionts
Source: Front Microbiol. 2022 Jul 28;13:927780. doi: 10.3389/fmicb.2022.927780 (PMC9366602; doi:10.3389/fmicb.2022.927780)
Supplement: Supplementary file 1 [file Data_Sheet_1.docx]

**Supporting Information**

**Traits along the Leaf Economics Spectrum structure communities of foliar endophytic symbionts**

Peter H. Tellez^1^, A. Elizabeth Arnold^2,3^, Ashton B. Leo^2^, Kaoru Kitajima^4,5^, and Sunshine A. Van Bael^1,4^

^1^*Department of Ecology and Evolutionary Biology, Tulane University, New Orleans LA, 70118, U.S.A.; ^2^School of Plant Sciences, University of Arizona, Tucson, AZ 85721, U.S.A.; ^3^Department of Ecology and Evolutionary Biology, University of Arizona, Tucson, AZ 85721, U.S.A.; ^4^Smithsonian Tropical Research Institute, Apartado Postal 0843-03092, Balboa, Ancon, Republic of Panama.; ^5^Division of Forest and Biomaterial Science, Graduate School of Agriculture, Kyoto University, Japan.*

**Corresponding author**:

Peter H. Tellez, 6823 St. Charles Ave, New Orleans, LA 70118, [Ptellez@tulane.edu](mailto:Ptellez@tulane.edu), 504.410.6939. Current address: 418 E. Glendale Ave, Alexandria, VA 22301.

The following Supporting Information is available for this article:

Figs. S1-S7

Tables S1-S10

**Supporting Information Figures S1-S6.**


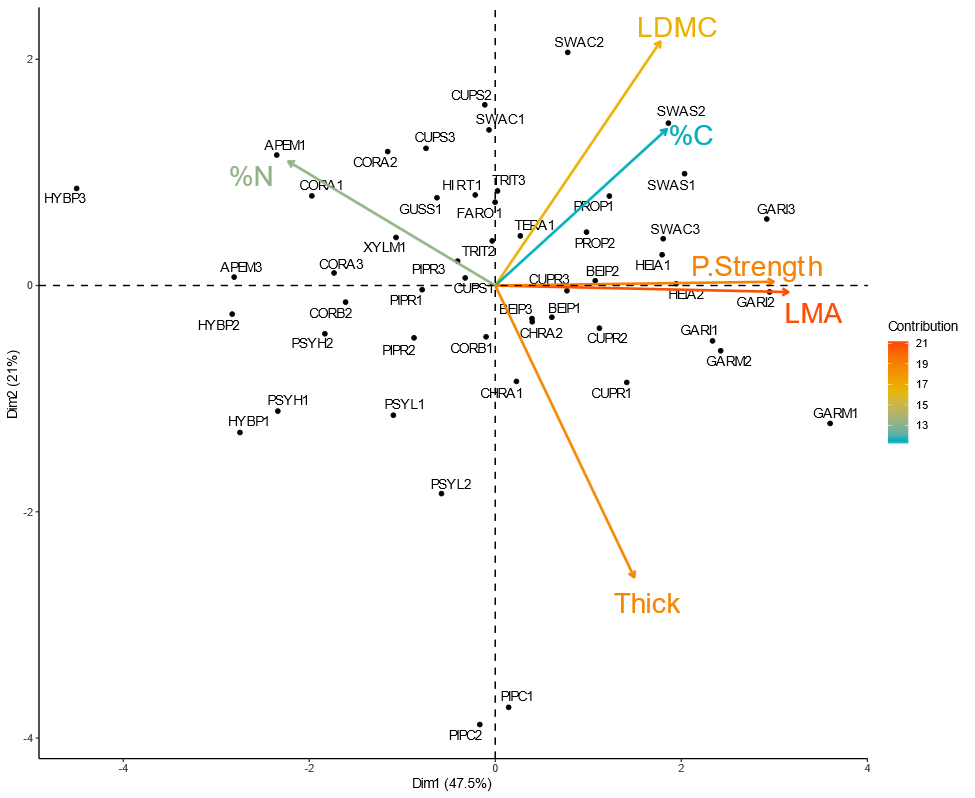


**Fig. S1.** Principal component analyses of six leaf functional traits of tree species sampled from Barro Colorado Island. Leaf mass per area (LMA, g m^-2^), leaf punch strength (P. Strength, log N mm^-1^), leaf thickness (Thick, log µm), leaf dry matter content (LDMC, g g^-1^), leaf carbon (%C, log %), leaf nitrogen (%N, log %). Legend shows the contribution of each trait to the principal component. For a list of tree species abbreviations, see Table 1 and Table S1.


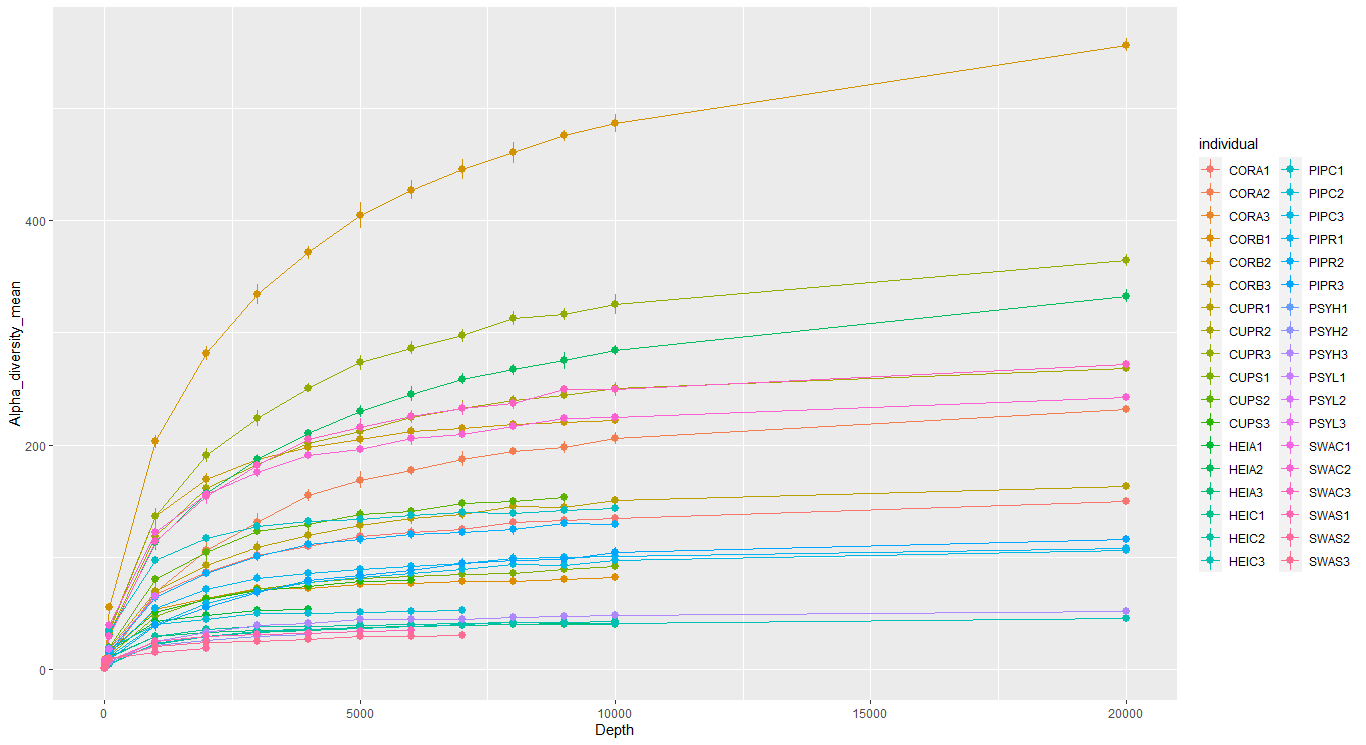


**Fig. S2.** Rarefaction curves of fungal endophytes detected from healthy leaves of 36 tree individuals in 14 plant species at Barro Colorado Island, AZ. OTUs were assigned based 95% sequence similarity of the ITS1 rDNA region.


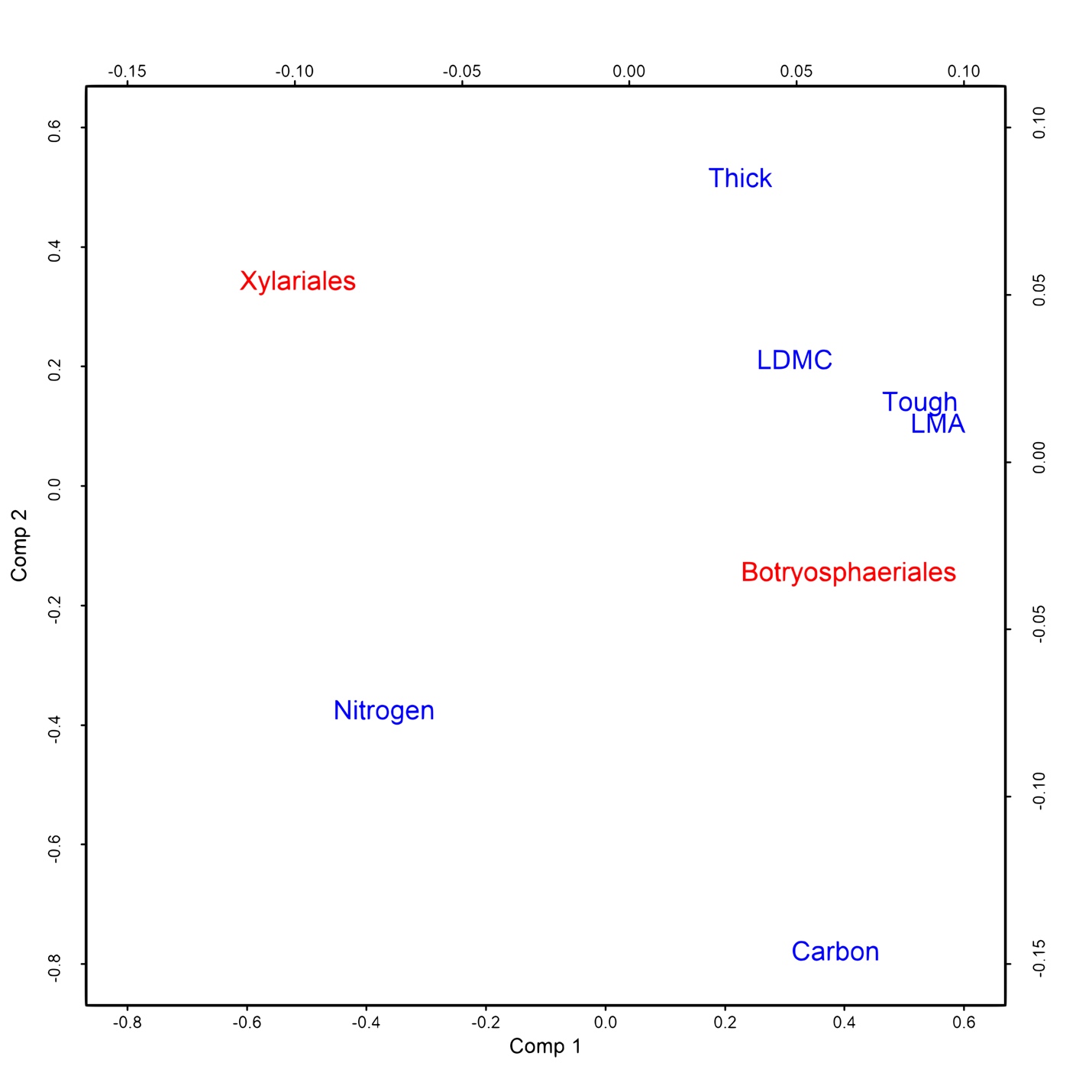


**Fig. S3.** Partial least squares regression (PLSR) loading plot identified associations among Xylariales, Botryosphaeriales, and leaf functional traits. Nitrogen: foliar %N; Carbon: foliar %C; LMDC: leaf dry matter content; Tough: leaf punch strength; LMA: leaf mass per area; Thick: leaf thickness. Variables that are closer together generally mean positive associations.


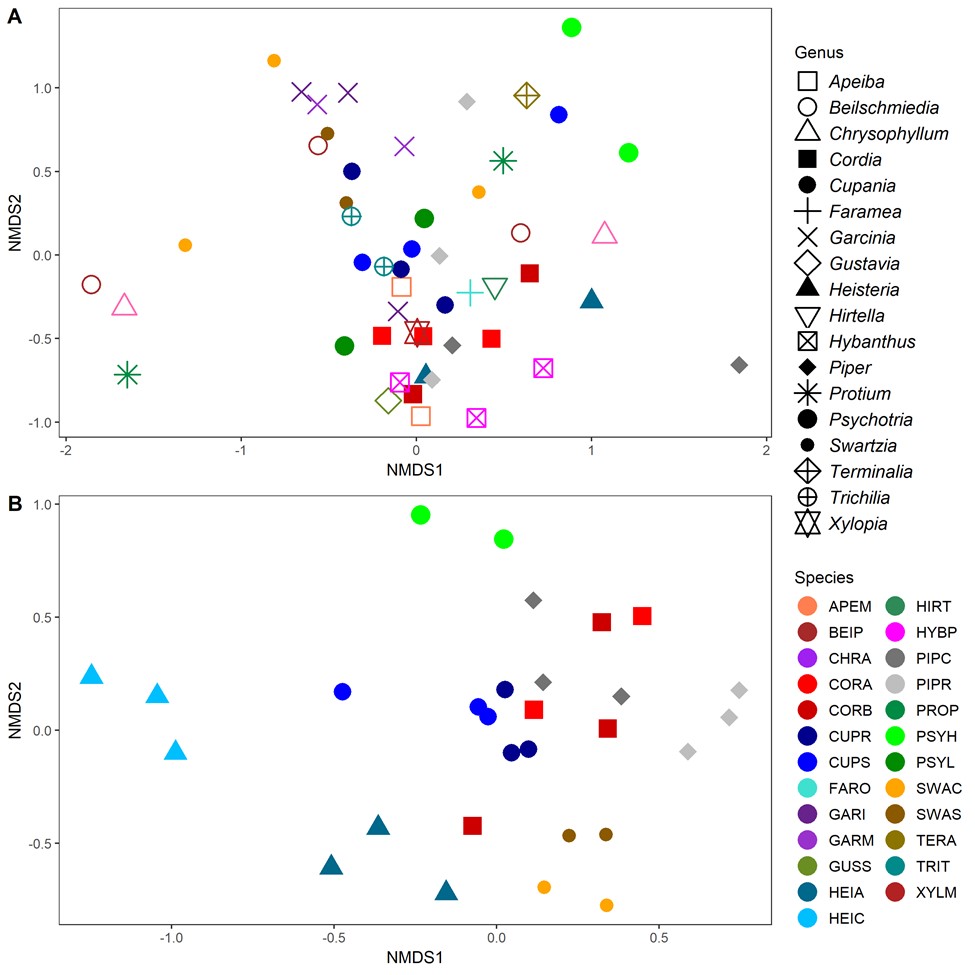


**Fig. S4.** Before constraining for leaf traits, endophyte community composition differed among host species and genera. Non-metric multidimensional scaling (NMDS) ordination of fungal endophyte community composition from (A) culture-based, and (B) culture-free endophyte surveys. Each point represents an endophyte community sampled from host tree, symbols represent host genera, and colors represent host species (for a list of abbreviations, see Table S1). Color-filled shapes represent tree taxa sampled for both culture-based and culture-free surveys.


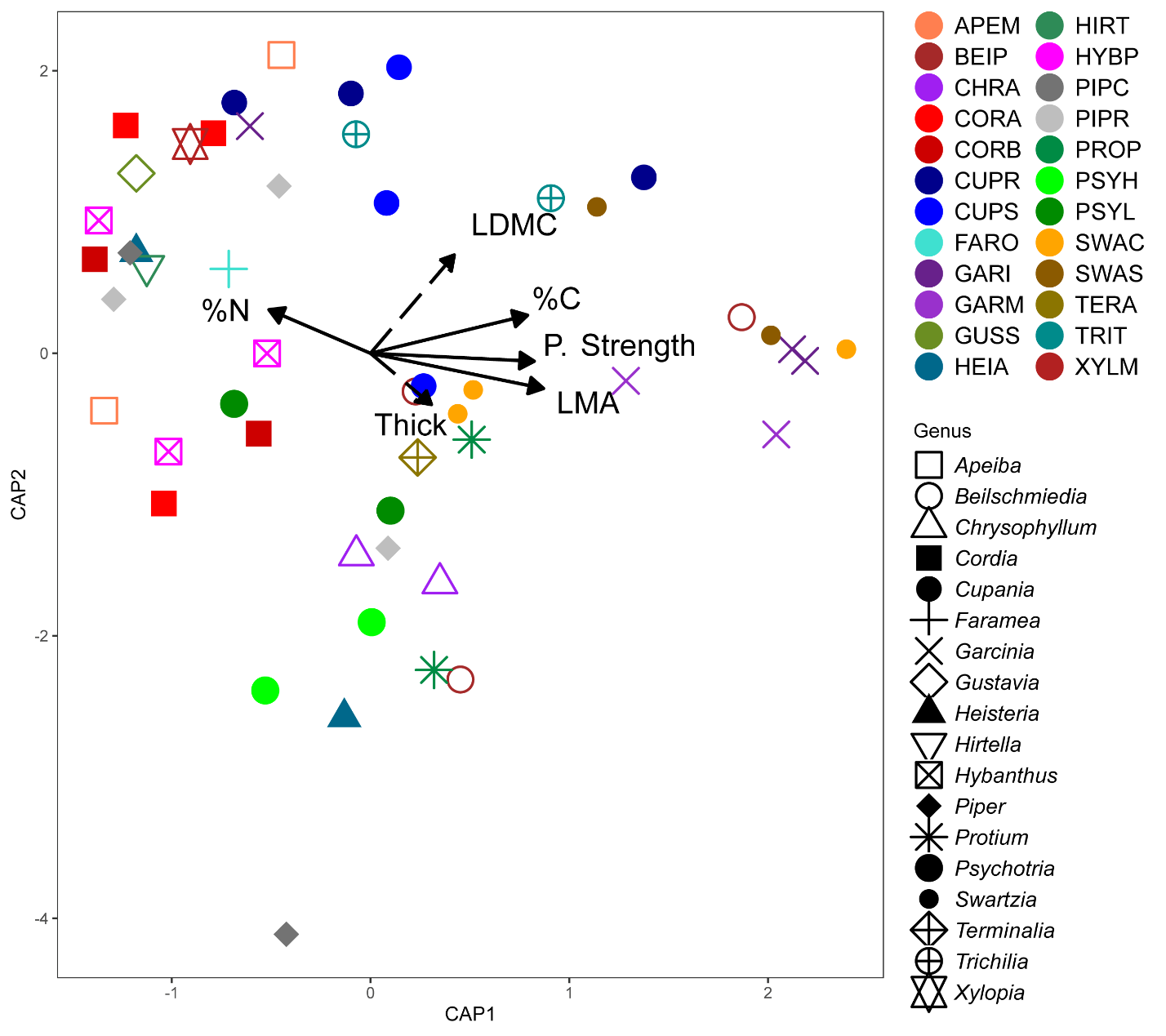
**Fig. S5. Culture-based endophyte community composition was associated with leaf traits from the leaf economics spectrum.** Endophyte community variation within and between 25 host species in 18 genera (n = 51), from dbRDA models constrained by leaf traits. Solid arrows represent significant associations (P < 0.05). Each point represents an endophyte community sampled from one host tree; colors represent host species; symbols represent host genus (for a list of host species abbreviations, see Table S1).

**
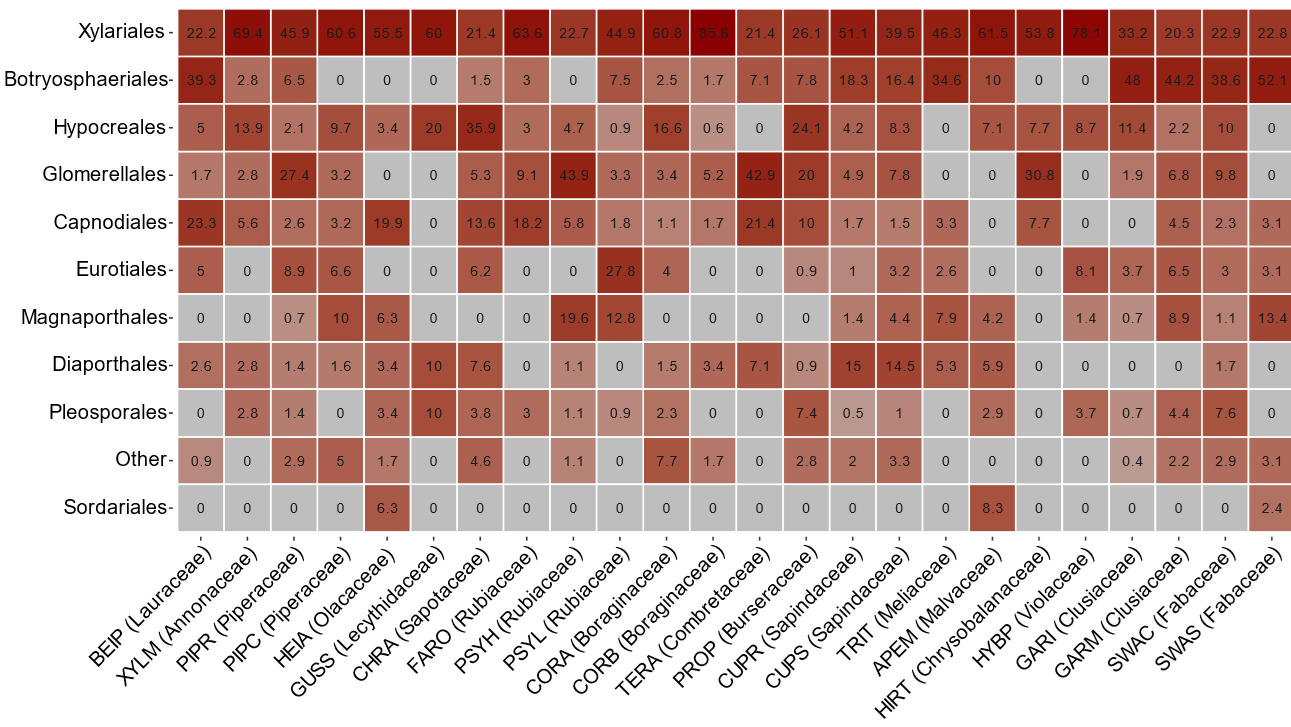
**

**Fig. S6.** Heat-map of percent relative abundance of fungal orders (left) by host species (bottom; for a list of abbreviations, see Table S1) from the culture-based survey. Numbers within map are the mean percent relative abundance of fungal orders within a host species.


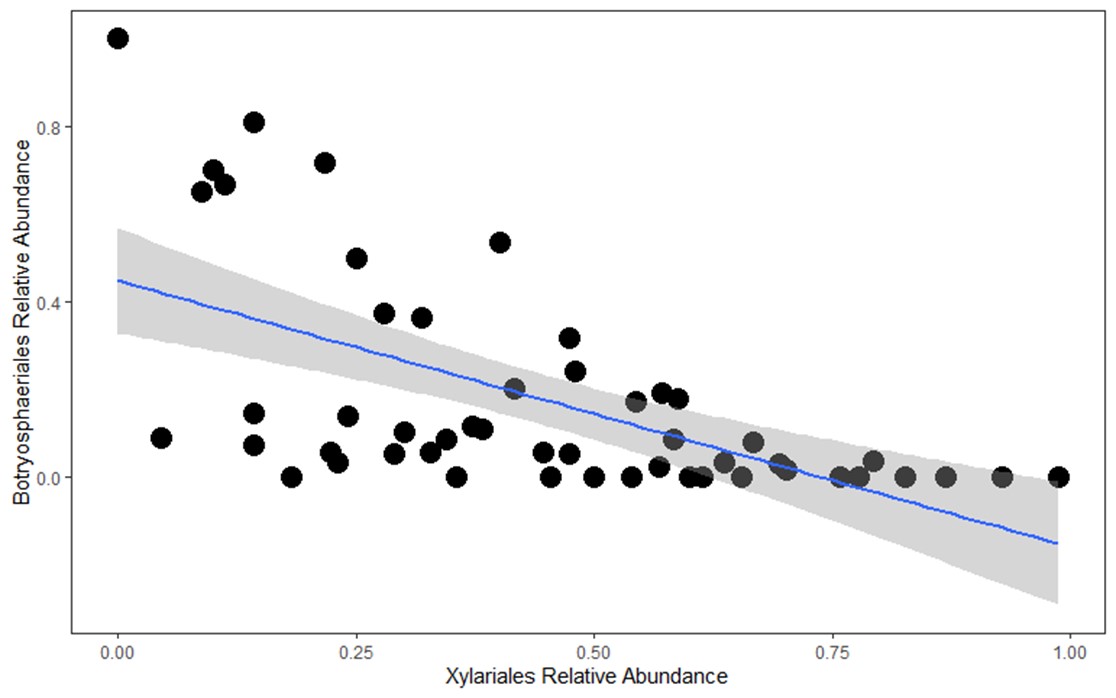


**Fig. S7.** Association between the relative abundance of fungal orders Xylariales and Botryosphaeriales from the culture-based survey. Pearson correlation coefficient test: F = 26.73, df = 49, P < 0.001, Adj. R^2^ = 0.34. Grey bands represent one standard error of the mean

**Supporting Information Tables S1-S9.**

**Table S1.** Results of culture-based survey of fungal endophytes associated with 30 woody plant species in the forest understory of Barro Colorado Island, Panama. Columns indicate plant family; plant species; species code; number of trees used for (culture-based survey/ culture-free survey); number of fungal isolates cultured; richness of OTU (number of different OTU in culture-based survey / culture-free survey) analyses; isolation frequency (mean ± SE, proportion of segments per individual from which a fungus was isolated in culture); and diversity (Fisher’s α; mean for individuals per species ± SE); NA = Endophyte diversity could not be calculated due to sampled trees yielding < 9 OTU.

| Family | Species | Code | No. | Isolates | Richness | Isolation frequency | Fisher’s alpha |
| --- | --- | --- | --- | --- | --- | --- | --- |
| Tiliaceae | *Apeiba membranacea* | APEM | 3/- | 68 | 21 | 0.24 ± 0.03 | 13.3 ± 2.66 |
| Lauraceae | *Beilschmiedia pendula* | BEIP | 3/- | 164 | 44 | 0.60 ± 0.19 | 10.9 ± 5.19 |
| Sapotaceae | *Chrysophyllum argenteum* | CHRA | 3/- | 108 | 43 | 0.37 ± 0.29 | 26.2 ±2.79 |
| Sapotaceae | *Chrysophyllum caimito* | CHRC | 1/- | 5 | 3 | 0.05 | NA |
| Boraginaceae | *Cordia alliodora** | CORA | 3/2 | 141 | 41/262 | 0.49 ± 0.09 | 10.9± 2.70 |
| Boraginaceae | *Cordia bicolor** | CORB | 3/3 | 118 | 22/644 | 0.40 ± 0.23 | 11.5 ± 8.62 |
| Sapindaceae | *Cupania rufescens** | CUPR | 3/- | 234 | 42/581 | 0.81 ± 0.10 | 11.58 ± 1.38 |
| Sapindaceae | *Cupania seemannii** | CUPS | 3/3 | 175 | 47/280 | 0.61 ± 0.16 | 14.9 ± 4.84 |
| Rubiaceae | *Faramea occidentalis* | FARO | 1/- | 38 | 19 | 0.40 | 19.4 |
| Clusiaceae | *Garcinia intermedia*^1^* | GARI | 3/- | 142 | 30 | 0.49 ± 0.22 | 5.68 ± 2.05 |
| Clusiaceae | *Garcinia madruno*^1^* | GARM | 3/- | 56 | 19 | 0.19 ± 0.07 | 7.85 ± 1.135 |
| Lecythidaceae | *Gustavia superba* | GUSS | 2/- | 35 | 10 | 0.12 ± 0.02 | NA |
| Olacaeae | *Heisteria acuminata** | HEIA | 3/3 | 72 | 21/304 | 0.25 ± 0.13 | 31.4 ± 22.4 |
| Olacaeae | *Heisteria concinna*^1^* | HEIC | 3/3 | 21 | 8/113 | 0.07 ± 0.03 | NA |
| Chrysobalanaceae | *Hirtella americana* | HIRA | 3/- | 111 | 30 | 0.39 ± 0.15 | 13.8 ± 4.83 |
| Chrysobalanaceae | *Hirtella triandra* | HIRT | 2/- | 17 | 6 | 0.09 ± 0.06 | NA |
| Violaceae | *Hybanthus prunifolius* | HYBP | 3/- | 88 | 33 | 0.32 ± 0.04 | 34.74 ± 16.1 |
| Salicaceae | *Laetia thamnia* | LAET | 2/- | 18 | 12 | 0.09 ± 0.01 | NA |
| Piperaceae | *Piper cordulatum** | PIPC | 3/3 | 52 | 18/263 | 0.18 ± 11.7 | 12.5 ± 6.31 |
| Piperaceae | *Piper reticulatum** | PIPR | 3/3 | 115 | 30/289 | 0.40 ± 0.10 | 6.99 ± 3.05 |
| Burseraceae | *Protium panamense* | PROP | 2/- | 111 | 34 | 0.58 ± 0.42 | 17.2 ± 6.81 |
| Rubiaceae | *Psychotria horizontalis** | PSYH | 3/2 | 109 | 43/75 | 0.38 ± 0.11 | 9.56 ± 1.68 |
| Rubiaceae | *Psychotria limonensis** | PSYL | 3/- | 105 | 29 | 0.42 ± 0.13 | 10.9 ± 4.11 |
| Fabaceae | *Swartzia simplex* var. *continentalis** | SWAS | 3/3 | 59 | 12/369 | 0.31 ± 0.10 | 4.3 ± 2.21 |
| Fabaceae | *Swartzia simplex* var*. grandiflora** | SWAC | 3/3 | 132 | 47/60 | 0.47 ± 0.24 | 17.3 ± 9.12 |
| Clusiaceae | *Symphonia globulifera* | SYMG | 1/- | 12 | 2 | 0.13 | NA |
| Combretaceae | *Terminalia amazonia* | TERA | 2/- | 29 | 11 | 0.15 ± 0.06 | NA |
| Malvaceae | *Theobroma cacao* | THEC | 2/- | 23 | 11 | 0.12 ± 0.02 | NA |
| Meliaceae | *Trichilia tuberculata* | TRIT | 3/- | 57 | 15 | 0.20 ± 0.07 | 6.83 ± 0.15 |
| Annonaceae | *Xylopia macrantha* | XYLM | 2/- | 44 | 15 | 0.23 ± 0.17 | NA |

*Denotes tree species that were used for culture-free surveys.

^1^Tree individuals for these species did not meet the threshold of 4000 sequences for the culture-free surveys and were excluded from multivariate analyses.

**Table S2**. Leaf functional traits for 30 woody plant species of Barro Colorado Island, Panama (mean ± SD): Leaf mass per area (LMA, g m^-2^); leaf dry-matter content (LDMC, g g^-1^); leaf punch strength (P. Strength, N mm^-1^); leaf thickness (Thick, µm); % Carbon; and % Nitrogen.

| Family | Species | LMA | LDMC | P. Strength | Thick | %C | %N |
| --- | --- | --- | --- | --- | --- | --- | --- |
| Tiliaceae | *Apeiba membranacea* | 26.1 ± 1.27 | 0.30 ± 0.07 | 0.93 ± 0.15 | 0.10 ± 0.01 | 55.0 ± 17.3 | 2.75 ± 0.72 |
| Lauraceae | *Beilschmiedia pendula* | 70.1 ± 1.75 | 0.32 ± 0.02 | 1.93 ± 0.14 | 0.20 ± 0.01 | 49.3 ± 1.40 | 1.95 ± 0.01 |
| Sapotaceae | *Chrysophyllum argenteum* | 61.7 ± 2.20 | 0.35 ± 0.02 | 2.65 ± 0.22 | 0.20 ± 0.01 | 43.4 ± 1.04 | 2.52 ± 0.61 |
| Sapotaceae | *Chrysophyllum cainito* | 63.3 | 0.50 | 2.67 | 0.17 | NA | NA |
| Boraginaceae | *Cordia alliodora* | 46.5 ± 1.86 | 0.43 ± 0.10 | 1.35 ± 0.14 | 0.16 ± 0.01 | 42.7 ± 0.45 | 3.11 ± 0.34 |
| Boraginaceae | *Cordia bicolor* | 49.9 ± 10.0 | 0.42 ± 0.03 | 2.04 ± 0.41 | 0.23 ± 0.03 | 42.8 ± 2.52 | 3.04 ± 0.76 |
| Sapindaceae | *Cupania rufescens* | 58.2 ± 2.87 | 0.43 ± 0.01 | 2.40 ± 0.14 | 0.27 ± 0.03 | 47.6 ± 0.13 | 1.96 ± 0.11 |
| Sapindaceae | *Cupania seemannii* | 48.5 ± 3.74 | 0.42 ± 0.08 | 1.77 ± 0.08 | 0.14 ± 0.04 | 47.2 ± 0.89 | 2.28 ± 0.31 |
| Rubiaceae | *Faramea occidentalis* | 54.9 | 0.46 | 2.32 | 0.16 | 43.9 | 2.32 |
| Clusiaceae | *Garcinia intermedia* | 83.8 ± 5.02 | 0.46 ± 0.04 | 3.14 ± 0.08 | 0.26 ± 0.03 | 50.4 ± 0.97 | 1.65 ± 0.31 |
| Clusiaceae | *Garcinia madruno* | 82.8 ± 5.35 | 0.41 ± 0.03 | 4.50 ± 0.83 | 0.25 ± 0.04 | 46.1 ± 0.76 | 1.44 ± 0.35 |
| Lecythidaceae | *Gustavia superba* | 45.1 ± 1.72 | 0.34 ± 0.01 | 2.53 ± 0.24 | 0.16 ± 0.01 | 45.0 ± 1.83 | 3.32 ± 0.15 |
| Olacaeae | *Heisteria acuminata* | 82.4 ± 3.14 | 0.40 ± 0.01 | 2.53 ± 0.24 | 0.16 ± 0.01 | 45.0 ± 1.83 | 3.32 ± 0.15 |
| Olacaeae | *Heisteria concinna* | 78.4 ± 2.58 | 0.41 ± 0.03 | 3.93 ± 0.31 | 0.21 ± 0.01 | 50.6 ± 0.59 | 2.13 ± 0.27 |
| Chrysobalanaceae | *Hirtella americana* | 40.6 ± 14.8 | 0.48 ± 0.09 | 1.69 ± 0.31 | NA | 47.8 ± 6.53 | 2.32 ± 1.01 |
| Chrysobalanaceae | *Hirtella triandra* | 44.9 ± 0.56 | 0.55 ± 0.00 | 1.63 ± 0.38 | 0.17 ± 0.01 | 42.1 ± 0.44 | 2.06 ± 0.01 |
| Violaceae | *Hybanthus prunifolius* | 24.4 ± 3.50 | 0.25 ± 0.03 | 1.02 ± 0.39 | 0.15 ± 0.02 | 42.8 ± 1.09 | 3.09 ± 1.44 |
| Salicaceae | *Laetia thamnia* | 60.5 ± 1.44 | 0.37 ± 0.01 | 2.78 ± 0.33 | 0.19 ± 0.03 | 43.8 ± 0.07 | 2.11 ± 0.02 |
| Piperaceae | *Piper cordulatum* | 62.8 ± 4.14 | 0.22 ± 0.03 | 2.01 ± 0.14 | 0.39 ± 0.01 | 40.5 ± 0.34 | 2.29 ± 0.16 |
| Piperaceae | *Piper reticulatum* | 60.3 ± 1.28 | 0.40 ± 0.04 | 2.06 ± 0.21 | 0.15 ± 0.00 | 38.9 ± 1.05 | 2.26 ± 0.14 |
| Burseraceae | *Protium panamense* | 79.5 ± 8.43 | 0.44 ± 0.01 | 2.20 ± 0.03 | 0.16 ± 0.00 | 46.4 ± 0.37 | 1.99 ± 0.28 |
| Rubiaceae | *Psychotria horizontalis* | 40.4 ± 1.02 | 0.22 ± 0.01 | 1.37 ± 0.20 | 0.19 ± 0.02 | 45.2 ± 3.37 | 2.77 ± 0.43 |
| Rubiaceae | *Psychotria limonensis* | 50.4 ± 1.30 | 0.18 ± 0.00 | 2.16 ± 0.36 | 0.20 ± 0.04 | 43.1 ± 0.92 | 2.13 ± 0.14 |
| Fabaceae | *Swartzia simplex* var. *continentalis* | 82.9 ± 2.89 | 0.49 ± 0.01 | 4.48 ± 0.25 | 0.19 ± 0.01 | 48.6 ± 1.14 | 2.75 ± 0.28 |
| Fabaceae | *Swartzia simplex* var. *grandiflora* | 62.1 ± 6.17 | 0.46 ± 0.06 | 3.29 ± 0.14 | 0.16 ± 0.02 | 47.9 ± 2.09 | 2.67 ± 1.27 |
| Clusiaceae | *Symphonia globulifera* | 52.1 | 0.33 | 1.64 | 0.24 | NA | NA |
| Combretaceae | *Terminalia amazonia* | 56.8 ± 0.19 | 0.40 ± 0.00 | 1.89 ± 0.06 | 0.21 ± 0.05 | 47.3 ± 0.53 | 1.85 ± 0.18 |
| Malvaceae | *Theobroma cacao* | 43.9 ± 3.81 | 0.40 ± 0.03 | 1.93 ± 0.10 | 0.17 ± 0.00 | 45.8 ± 0.41 | 2.10 ± 0.38 |
| Meliaceae | *Trichilia tuberculata* | 63.2 ± 3.27 | 0.41 ± 0.02 | 1.92 ± 0.24 | 0.18 ± 0.00 | 47.7 ± 0.85 | 2.46 ± 0.19 |
| Annonaceae | *Xylopia macrantha* | 44.8 ± 2.39 | 0.41 ± 0.04 | 1.86 ± 0.15 | 0.17 ± 0.00 | 46.3 ± 3.04 | 2.51 ± 0.48 |

**Table S3.** Results of bioassays evaluating cellulase, chitinase, and protease activity. N, negative for bioassay activity; P, positive for bioassay activity; NT, not tested.

| Isolate | Fungal Order | Host Family | Host Code | Cellulase | Chitinase | Protease |
| --- | --- | --- | --- | --- | --- | --- |
| PT0029 | Xylariales | Boraginaceae | CORB2 | N | P | P |
| PT0061 | Xylariales | Boraginaceae | CORB3 | P | P | N |
| PT0101 | Xylariales | Boraginaceae | CORB3 | N | P | P |
| PT0159 | Xylariales | Boraginaceae | CORA1 | P | P | P |
| PT0173 | Botryosphaeriales | Boraginaceae | CORA1 | N | P | N |
| PT0188 | Xylariales | Boraginaceae | CORA2 | N | P | P |
| PT0204 | Xylariales | Boraginaceae | CORA2 | P | P | P |
| PT0207 | Xylariales | Boraginaceae | CORA2 | N | P | N |
| PT0257 | Botryosphaeriales | Clusiaceae | GARM1 | N | P | N |
| PT0262 | Botryosphaeriales | Clusiaceae | GARM1 | N | P | N |
| PT0267 | Botryosphaeriales | Clusiaceae | GARM1 | N | N | N |
| PT0268 | Xylariales | Clusiaceae | GARM1 | N | N | P |
| PT0301 | Botryosphaeriales | Clusiaceae | GARM3 | N | P | N |
| PT0311 | Botryosphaeriales | Clusiaceae | GARI1 | N | N | N |
| PT0313 | Botryosphaeriales | Clusiaceae | GARI1 | N | P | N |
| PT0315 | Xylariales | Clusiaceae | GARI1 | N | N | P |
| PT0347 | Botryosphaeriales | Clusiaceae | GARI2 | N | P | N |
| PT0367 | Botryosphaeriales | Clusiaceae | GARI2 | N | P | N |
| PT0386 | Xylariales | Clusiaceae | GARI3 | N | P | P |
| PT0394 | Xylariales | Clusiaceae | GARI3 | N | P | P |
| PT0417 | Xylariales | Clusiaceae | GARI3 | P | P | P |
| PT0425 | Xylariales | Clusiaceae | GARI3 | N | N | P |
| PT0427 | Xylariales | Clusiaceae | GARI3 | P | N | P |
| PT0453 | Xylariales | Boraginaceae | CORA3 | P | P | P |
| PT0457 | Xylariales | Boraginaceae | CORB3 | N | P | N |
| PT0506 | Xylariales | Piperaceae | PIPR2 | N | P | N |
| PT0535 | Botryosphaeriales | Piperaceae | PIPR3 | N | P | N |
| PT0564 | Botryosphaeriales | Piperaceae | PIPR3 | N | P | N |
| PT0611 | Xylariales | Rubiaceae | PSYH1 | P | P | P |
| PT0645 | Botryosphaeriales | Rubiaceae | PSYH2 | N | N | N |
| PT0656 | Botryosphaeriales | Rubiaceae | PSYH3 | N | N | N |
| PT0661 | Xylariales | Rubiaceae | PSYH3 | P | P | P |
| PT0682 | Xylariales | Rubiaceae | PSYL1 | N | P | P |
| PT0687 | Xylariales | Rubiaceae | PSYL1 | N | P | P |
| PT0701 | Botryosphaeriales | Rubiaceae | PSYL2 | N | N | N |
| PT0707 | Xylariales | Rubiaceae | PSYL2 | N | N | P |
| PT0740 | Xylariales | Rubiaceae | PSYL3 | P | P | N |
| PT0783 | Botryosphaeriales | Fabaceae | SWAC1 | N | N | N |
| PT0808 | Xylariales | Fabaceae | SWAC2 | N | P | P |
| PT0855 | Xylariales | Fabaceae | SWAC3 | P | N | N |
| PT0926 | Botryosphaeriales | Fabaceae | SWAS1 | N | N | N |
| PT0928 | Botryosphaeriales | Fabaceae | SWAS1 | N | N | N |
| PT0932 | Botryosphaeriales | Fabaceae | SWAS1 | N | P | N |
| PT0935 | Xylariales | Fabaceae | SWAS1 | N | P | P |
| PT0943 | Botryosphaeriales | Fabaceae | SWAS3 | N | N | N |
| PT0963 | Xylariales | Fabaceae | SWAS3 | N | P | N |
| PT0970 | Xylariales | Piperaceae | PIPC1 | N | P | P |
| PT1004 | Xylariales | Piperaceae | PIPC2 | N | P | P |
| PT1031 | Botryosphaeriales | Olacaeae | HEIC2 | N | N | N |
| PT1041 | Xylariales | Olacaeae | HEIA1 | N | P | P |
| PT1042 | Xylariales | Olacaeae | HEIA1 | P | P | P |
| PT1045 | Xylariales | Olacaeae | HEIA1 | P | P | P |
| PT1047 | Xylariales | Olacaeae | HEIA1 | N | P | P |
| PT1056 | Xylariales | Olacaeae | HEIA2 | P | P | P |
| PT1123 | Xylariales | Sapindaceae | CUPS1 | P | P | N |
| PT1170 | Xylariales | Sapindaceae | CUPS2 | P | P | P |
| PT1175 | Botryosphaeriales | Sapindaceae | CUPS2 | N | P | N |
| PT1187 | Xylariales | Sapindaceae | CUPS2 | P | P | N |
| PT1201 | Xylariales | Sapindaceae | CUPS2 | N | P | N |
| PT1249 | Botryosphaeriales | Sapindaceae | CUPS3 | P | P | P |
| PT1279 | Xylariales | Sapindaceae | CUPS3 | P | P | P |
| PT1285 | Botryosphaeriales | Sapindaceae | CUPR1 | P | P | N |
| PT1292 | Xylariales | Sapindaceae | CUPR1 | P | P | N |
| PT1307 | Botryosphaeriales | Sapindaceae | CUPR1 | N | P | N |
| PT1316 | Xylariales | Sapindaceae | CUPR1 | P | P | P |
| PT1367 | Xylariales | Sapindaceae | CUPR2 | N | P | N |
| PT1388 | Xylariales | Sapindaceae | CUPR2 | N | P | N |
| PT1398 | Xylariales | Sapindaceae | CUPR2 | N | P | N |
| PT1406 | Xylariales | Sapindaceae | CUPR2 | P | P | P |
| PT1434 | Xylariales | Sapindaceae | CUPR3 | P | P | P |
| PT1443 | Xylariales | Sapindaceae | CUPR3 | N | P | P |
| PT1500 | Botryosphaeriales | Sapindaceae | CUPR3 | N | P | P |
| PT1611 | Xylariales | Meliaceae | TRIT3 | N | P | P |
| PT1616 | Xylariales | Meliaceae | TRIT3 | N | P | P |
| PT1647 | Xylariales | Rubiaceae | FARO3 | P | P | P |
| PT1649 | Xylariales | Rubiaceae | FARO3 | P | P | N |
| PT1698 | Xylariales | Tiliaceae | APEM1 | P | P | P |
| PT1756 | Xylariales | Lauraceae | BEIP1 | P | P | P |
| PT1772 | Xylariales | Lauraceae | BEIP1 | N | P | N |
| PT1774 | Xylariales | Combretaceae | TERA2 | P | P | N |
| PT1821 | Xylariales | Chrysobalanaceae | HIRT2 | N | P | P |
| PT1828 | Xylariales | Rubiaceae | FARO3 | P | P | P |
| PT1831 | Xylariales | Rubiaceae | FARO3 | N | N | P |
| PT1835 | Xylariales | Sapotaceae | CHRA2 | N | P | P |
| PT2010 | Botryosphaeriales | Meliaceae | TRIT2 | N | P | P |
| PT2011 | Botryosphaeriales | Meliaceae | TRIT2 | P | P | P |
| PT2014 | Xylariales | Meliaceae | TRIT2 | P | P | P |
| PT2104 | Xylariales | Piperaceae | PIPR3 | N | N | NT |
| PT2117 | Xylariales | Piperaceae | PIPR3 | P | P | P |
| PT2209 | Xylariales | Chrysobalanaceae | HIRA2 | N | P | P |
| PT2236 | Botryosphaeriales | Chrysobalanaceae | HIRA3 | N | P | N |
| PT2239 | Xylariales | Chrysobalanaceae | HIRA3 | N | P | P |
| PT2305 | Botryosphaeriales | Piperaceae | PIPR1 | N | P | N |
| PT2311 | Xylariales | Sapotaceae | CHRC1 | P | NT | NT |
| PT2319 | Xylariales | Violaceae | HYBP1 | P | P | P |
| PT2379 | Xylariales | Violaceae | HYBP3 | P | P | P |
| PT2383 | Xylariales | Violaceae | HYBP3 | N | P | P |
| PT2389 | Xylariales | Violaceae | HYBP3 | N | N | P |
| PT2434 | Xylariales | Burseraceae | PROP3 | N | N | N |
| PT2749 | Botryosphaeriales | Annonaceae | XYLM2 | N | N | N |
| PT2755 | Xylariales | Annonaceae | XYLM2 | N | N | P |
| PT2777 | Xylariales | Annonaceae | XYLM2 | N | P | P |
| PT2778 | Xylariales | Annonaceae | XYLM2 | N | P | P |
| PT2854 | Xylariales | Lauraceae | BEIP3 | N | P | P |
| PT2888 | Xylariales | Violaceae | HYBP3 | N | P | P |
| PT2896 | Botryosphaeriales | Lauraceae | BEIP2 | N | N | N |

**Table S4.** Results of fungal antagonism assays. The first four columns indicate strain ID, clade (X, Xylariales; B, Botryosphaeriales; number, operational taxonomic unit at 95% similarity), order, and activity (both = chitinase and protease activity observed, as shown in Table S9; none = neither chitinase nor protease activity observed, as shown in Table S9. We did not consider cellulase activity in selecting strains for these assays because we anticipated that cellulase activity would not be relevant against a fungal pathogen, relative to chitinase and protease activity). The last two columns indicate % inhibition of the pathogen by the endophyte, or % inhibition of the endophyte by the pathogen. Inhibition was measured as the reduction in colony radius in response to the co-plated isolate, relative to the colony radius in the absence of the co-plated isolate. Percent inhibition was calculated as inhibition / colony radius in the absence of the co-plated isolate. A negative value indicates growth enhancement.

| Strain | Clade | Order | Activity | % inhibition of *Fusarium* sp. by endophyte | % inhibition of endophyte by *Fusarium* sp. |
| --- | --- | --- | --- | --- | --- |
| PT0188 | X1 | Xylariales | both | 26.2% | 69.7% |
| PT0386 | X10 | Xylariales | both | 32.3% | 54.5% |
| PT0417 | X1 | Xylariales | both | 21.3% | 0.0% |
| PT0611 | X2 | Xylariales | both | 32.3% | 28.0% |
| PT0661 | X8 | Xylariales | both | 31.1% | 58.6% |
| PT1004 | X11 | Xylariales | both | 32.3% | 23.1% |
| PT1249 | B3 | Botryosphaeriales | both | 33.3% | 63.6% |
| PT1434 | X5 | Xylariales | both | 37.5% | -10.0% |
| PT1500 | B4 | Botryosphaeriales | both | 25.8% | 64.1% |
| PT2011 | B2 | Botryosphaeriales | both | 28.1% | 9.1% |
| PT2778 | X1 | Xylariales | both | 25.8% | 29.0% |
| PT0311 | B1 | Botryosphaeriales | none | 24.2% | 41.2% |
| PT0656 | B5 | Botryosphaeriales | none | 12.9% | 66.7% |
| PT0701 | B5 | Botryosphaeriales | none | 20.6% | 87.5% |
| PT0783 | B2 | Botryosphaeriales | none | 26.2% | 60.0% |
| PT1031 | B3 | Botryosphaeriales | none | 20.6% | 44.4% |
| PT2434 | X6 | Xylariales | none | 36.4% | 58.1% |

**Table S5.** Kruskal-Wallis tests for differences in endophyte abundance and diversity among host species and genera for the culture-based survey.

|  | **Chi-squared** | **Df** | ***P*** |
| --- | --- | --- | --- |
| **Endophyte Abundance** |  |  |  |
| *Species* | 16.9 | 23 | 0.81 |
| *Genus* | 13.6 | 17 | 0.69 |
| **Endophyte Diversity** |  |  |  |
| *Species* | 18.2 | 23 | 0.74 |
| *Genus* | 15.6 | 17 | 0.55 |

**Table S6.** Summary of PLSR variable importance on projections (VIP) values for the first 2 latent variables (LV). Importance of variables are as follows: highly influential variables (> 1 VIP), moderately influential (1 – 0.7 VIP), and less influential (< 0.7 VIP).

|  | **Culture-based Endophyte Abundance** | | **Culture-based Endophyte Diversity** | | **Culture-free**  **Endophyte Richness** | |
| --- | --- | --- | --- | --- | --- | --- |
| **Leaf Trait** | *LV1* | *LV2* | *LV1* | *LV2* | *LV1* | *LV2* |
| LMA | 0.06 | 0.69 | 1.24 | 1.12 | 1.13 | 0.94 |
| LDMC | 1.07 | 0.98 | 1.40 | 1.40 | 0.04 | 0.90 |
| P. Strength | 1.37 | 1.21 | 1.18 | 1.07 | 1.03 | 1.09 |
| Thick | 0.49 | 0.88 | 0.64 | 0.57 | 0.48 | 0.39 |
| %C | 1.16 | 1.07 | 0.14 | 0.83 | 1.88 | 1.59 |
| %N | 1.15 | 1.06 | 0.79 | 0.76 | 0.19 | 0.61 |

**Table S7.** Mantel tests for culture-based and culture-free endophyte surveys showing the relationships between host phylogenetic relatedness and endophyte community composition; physical proximity of hosts to each other and endophyte community composition; and host phylogeny and leaf functional traits.

|  | **Culture-based survey** | | **Culture-free survey** | |
| --- | --- | --- | --- | --- |
| **Tested relationships** | **R** | ***P*** | **R** | ***P*** |
| Host phylogeny vs endophyte community composition | 0.07 | 0.30 | 0.05 | 0.32 |
| Host proximity vs. endophyte community composition | -0.04 | 0.71 | 0.04 | 0.65 |
| Host phylogeny vs host leaf functional traits | 0.01 | 0.87 | 0.09 | 0.71 |

**Table S8.** Relationships between leaf functional traits and endophyte community composition as determined by PERMANOVA marginal tests.

|  | **Culture-based survey** | | | **Culture-free survey** | | |
| --- | --- | --- | --- | --- | --- | --- |
| **Leaf trait** | **F** | ***P*** | **R^2^** | **F** | ***P*** | **R^2^** |
| LMA | 4.03 | 0.002 | 0.076 | 1.72 | 0.001 | 0.065 |
| LMDC | 1.54 | 0.068 | 0.031 | 1.26 | 0.071 | 0.046 |
| Thick | 1.32 | 0.164 | 0.025 | 0.94 | 0.604 | 0.031 |
| P. Strength | 3.59 | 0.003 | 0.068 | 2.16 | 0.001 | 0.093 |
| %C | 3.40 | 0.002 | 0.065 | 1.87 | 0.001 | 0.080 |
| %N | 1.57 | 0.049 | 0.033 | 1.13 | 0.047 | 0.054 |

**Table S9.** Partial-dbRDA results showing proportion of variation in endophyte community composition explained by host species and leaf traits in culture-based and culture-free surveys.

|  | **Culture-based survey** | | **Culture-free survey** | |
| --- | --- | --- | --- | --- |
|  | Variance explained by leaf traits (constrained) when controlling for host species (conditional) | Variance explained by host species (constrained) when controlling for leaf traits (conditional) | Variance explained by leaf traits (constrained) when controlling for host species (conditional) | Variance explained by host species (constrained) when controlling for leaf traits (conditional) |
| **Total** | 1.000 | 1.000 | 1.000 | 1.000 |
| **Constrained** | 0.1043 | 0.4783 | 0.0865 | 0.4246 |
| **Conditional** | 0.5526 | 0.1787 | 0.7228 | 0.3847 |
| **Unconstrained** | 0.3915 | 0.3915 | 0.1933 | 0.1933 |
| **Imaginary** | -0.0485 | -0.0485 | -0.0027 | -0.0027 |

**Table S10.** Pearson correlation coefficient tests for associations between leaf functional traits and the relative abundance of fungal orders Xylariales and Botryosphaeriales in the culture-based survey.

|  | **Xylariales** | | | **Botryosphaeriales** | | |
| --- | --- | --- | --- | --- | --- | --- |
| **Leaf trait** | **F** | ***P*** | **R^2^** | **F** | ***P*** | **R^2^** |
| LMA | 17.5 | <0.01 | 0.25 | 14.9 | <0.01 | 0.22 |
| LDMC | 1.03 | 0.31 | 0.03 | 5.45 | 0.02 | 0.08 |
| Thick | 0.75 | 0.38 | 0.02 | 1.22 | 0.27 | 0.02 |
| P. Strength | 13.5 | <0.01 | 0.20 | 13.9 | <0.01 | 0.20 |
| %C | 12.1 | <0.01 | 0.18 | 13.7 | <0.01 | 0.20 |
| %N | 4.41 | 0.04 | 0.06 | 5.04 | 0.0 | 0.07 |
